# Distinct YY dinucleotide periodicity in adeno-associated virus DNA

**DOI:** 10.1016/j.ymthe.2026.07.003

**Published:** 2026-07-08

**Authors:** Conradin Baumgartl, Jonas Becker, Lena Naber, Man Xu, Emma Gerstmann, Julie Garcia Gonzalez-Calero, Joyce Suppacher, Sebastian Heß, Felix Bubeck, Dirk Grimm

**Affiliations:** 1 Department of Infectious Diseases/Virology, Section Viral Vector Technologies, Medical Faculty, Heidelberg University, BioQuant, Center for Integrative Infectious Diseases (CIID), 69120 Heidelberg, Germany; 2 AbbVie Deutschland GmbH & Co. KG, 67061 Ludwigshafen, Germany; 3 German Center for Infection Research (DZIF) and German Center for Cardiovascular Research (DZHK), Partner Site Heidelberg, 69120 Heidelberg, Germany; 4 Faculty of Engineering Sciences, Heidelberg University, 69120 Heidelberg, Germany

**Keywords:** adeno-associated virus, AAV, genome, periodicity, vectors

## Abstract

Dinucleotide periodicity is a hallmark of genome organization, yet its role in single-stranded (ss)DNA viruses remains poorly understood. Here, we systematically analyzed dinucleotide spacing patterns in adeno-associated virus (AAV) genomes and other viruses. Across 13 primate AAV serotypes, we identified a pronounced and highly conserved ∼15-bp periodicity specific to pyrimidine-pyrimidine (YY) dinucleotides and their reverse complements (RR). Comparative analyses across >25,000 viral sequences demonstrate that this 15-bp YY/RR periodicity is unique to the genus *Dependoparvovirus* and absent from other ssDNA viruses, satellite viruses, and helper viruses, which predominantly exhibit canonical ∼10- to 11-bp periodicities. Upon disruption of the YY/RR pattern using DNA family shuffling of AAV capsid genes, and subsequent iterative selection for viral production or cell entry, we found that the pattern is under positive selection. Selected sequences display increased periodicity alongside reduced sequence diversity, supporting a functional role for this genomic feature. Finally, engineered recombinant AAV genomes containing YY periodic motifs exhibit enhanced production and, for some designs, improved transduction efficiency, demonstrating that YY periodicity can modulate viral replication and infectivity. Our findings uncover a unique DNA-encoded signal in dependoparvoviruses that contributes to AAV fitness, expands our knowledge of virus biology, and has implications for vector engineering.

## Graphical abstract

**Figure:**
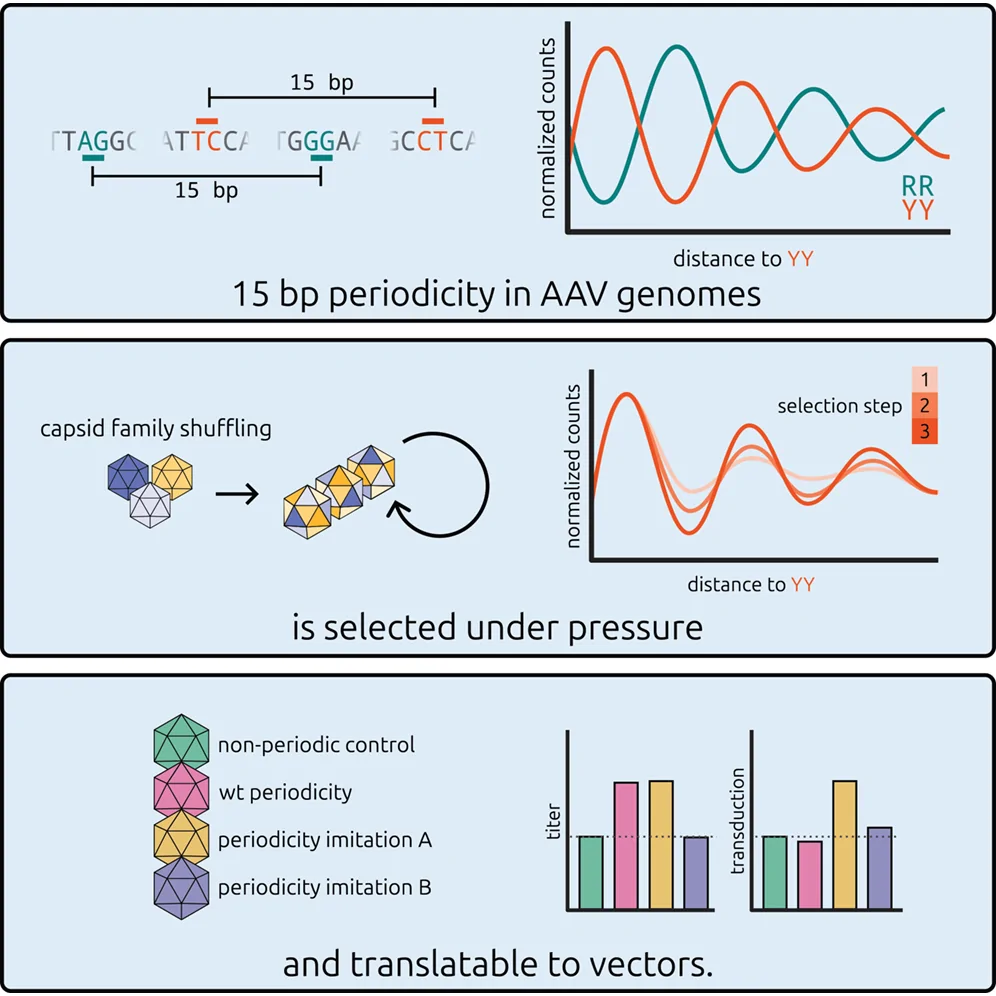

## Teaser

This work reveals a conserved ∼15-bp pyrimidine-pyrimidine dinucleotide periodicity within the genomes of adeno-associated viruses (AAVs) that is unique to *Dependoparvovirus*. The pattern is under positive selection, enhances viral production and infectivity, and represents a previously unrecognized DNA-encoded signal with implications for virus biology and AAV vector engineering.

## Introduction

Adeno-associated viruses (AAVs) are minute, non-enveloped, icosahedral viruses that package a single-stranded DNA (ssDNA) genome within their protein shell (capsid). They have been successfully developed and used for gene therapy approaches over the past four decades, culminating in multiple approved gene therapy products that utilize recombinant AAVs (rAAV) as their core ingredient. The success of AAV as a DNA delivery vector in gene therapy is based on its low immunogenicity, high transduction capability, and relative ease of manipulation.^1^ Nevertheless, multiple challenges prevent rAAV vectors from realizing their full potential, such as high seroprevalence in the human population^2^^,^^3^; low specificity in certain target cell populations^4^^,^^5^; limited long-term expression of delivered genes, especially in replicating cells^6^^,^^7^; or inefficient vector production.^8^ The last contributes to the high price tags of current gene therapeutics,^9^^,^^10^ which decrease the willingness of insurance providers to cover treatment costs, ultimately directly harming patients in need of a cure.

As replication-deficient viruses, AAVs require host and other viral factors to replicate efficiently, such as those provided by the name-giving adenovirus, which is most commonly used as a helper virus for rAAV manufacturing.^11^ The only indispensable AAV genetic *cis* elements necessary for DNA packaging are the inverted terminal repeat (ITR) sequences, i.e., 145-nt-long (in the AAV2 prototype) DNA stretches at the two ends of the viral genome. For viral vector production in cell culture, wild-type AAV and adenoviral helper genes are supplied in *trans* together with an ITR-flanked therapeutic or reporter cassette. The production occurs through packaging of the replicated viral DNA into pre-formed capsids with the help of the AAV proteins Rep52 and Rep40.^12^^,^^13^ Producing rAAV in this manner is often burdened by relatively high proportions of unwanted empty particles as compared with the production of AAV containing the wild-type genome, which results in much better full-to-empty particle ratios.^8^^,^^14^ Previously, multiple possible explanations for this observation have been proposed, such as a necessary coupling of replication and encapsidation or the presence of a *cis*-acting packaging signal outside of the ITR sequences.^8^^,^^15^

Periodicities in DNA sequences are described in all branches of life.^16^^,^^17^^,^^18^^,^^19^^,^^20^ These sequence periodicities can be rather inconspicuous, as they are usually detectable only when analyzing and averaging over multiple kilobases of sequence information. Prominent examples are the 3-bp periodicity in protein-coding sequences and the 10-bp periodicity in histone-wrapped DNA. Coding sequences preferentially contain the codon RNY (R, purine; N, any nucleotide; Y, pyrimidine), which has been linked to a putative primordial codon.^19^^,^^21^ This periodic characteristic is utilized in state-of-the-art gene-prediction algorithms to identify coding sequences within newly assembled genomes.^22^ In contrast to the 3-bp periodicity of coding sequences, the 10-bp periodicity of specific dinucleotides within eukaryotic genomes is linked to the facilitation of DNA-histone interactions. The 10-bp repeat mirrors the 10 bp of one complete turn of the B-DNA double helix. The 10-bp periodic presence of a dinucleotide produces perturbations on one side of the double helix, ultimately introducing a bend.^23^ This is thought to facilitate the winding of DNA around the histone core, and its local presence within genomes has been proposed as a possible nucleosome positioning code.^17^^,^^18^ The exact repeating dinucleotide can differ between species, as illustrated by, for instance, the CG dinucleotide in humans^24^ or the AA/TT dinucleotides in *Saccharomyces cerevisiae*.^25^

Surprisingly, sequence periodicities remain relatively understudied in viral genomes, likely due to the typically short length of these genomes that contrasts with the requirement to analyze multiple kilobases of sequence data to detect such patterns. Moreover, the evolution of periodicities in viral genomes has to compete with the pressure on viruses to optimize the information density within the size constraints of their genomes. Nonetheless, it is likely that viruses, too, have evolved genomic patterns to optimize processes critical for their life cycle. In fact, the 3-bp periodicity of coding sequences can be detected in viral sequences,^26^ but examples of larger-order periodicities are rare in the literature. For instance, there is evidence for specific dinucleotide patterns in bacteriophages, such as phi29 and *Erwinia* phages.^27^^,^^28^^,^^29^ Intriguingly, sequences of protein-VII-bound DNA in adenoviruses exhibit a dinucleotide periodicity with a shorter repeat length as compared with nucleosome-bound DNA sequences of around 6 bp. One possible explanation is that adenoviruses evolved this sequence pattern as an adaptation to enhance their DNA’s association with protein VII.^30^^,^^31^

In this work, we analyzed dinucleotide patterns within the genus *Dependoparvovirus* as well as in control and model organism genomes, leading to the discovery that AAVs possess a unique and pronounced ∼15-bp YY/RR dinucleotide pattern. We demonstrate that this pattern is under selection, and we provide evidence that it can influence viral DNA packaging and transduction, i.e., delivery and expression of virally encoded transgenes in target cells. In the future, the inclusion of this previously unknown wild-type sequence pattern in recombinant viral vector cassettes could be harnessed to optimize AAV-based human gene therapy vectors.

## Results

### AAV genomes exhibit a pronounced 15-bp YY/RR dinucleotide periodicity

**Figure 1:**
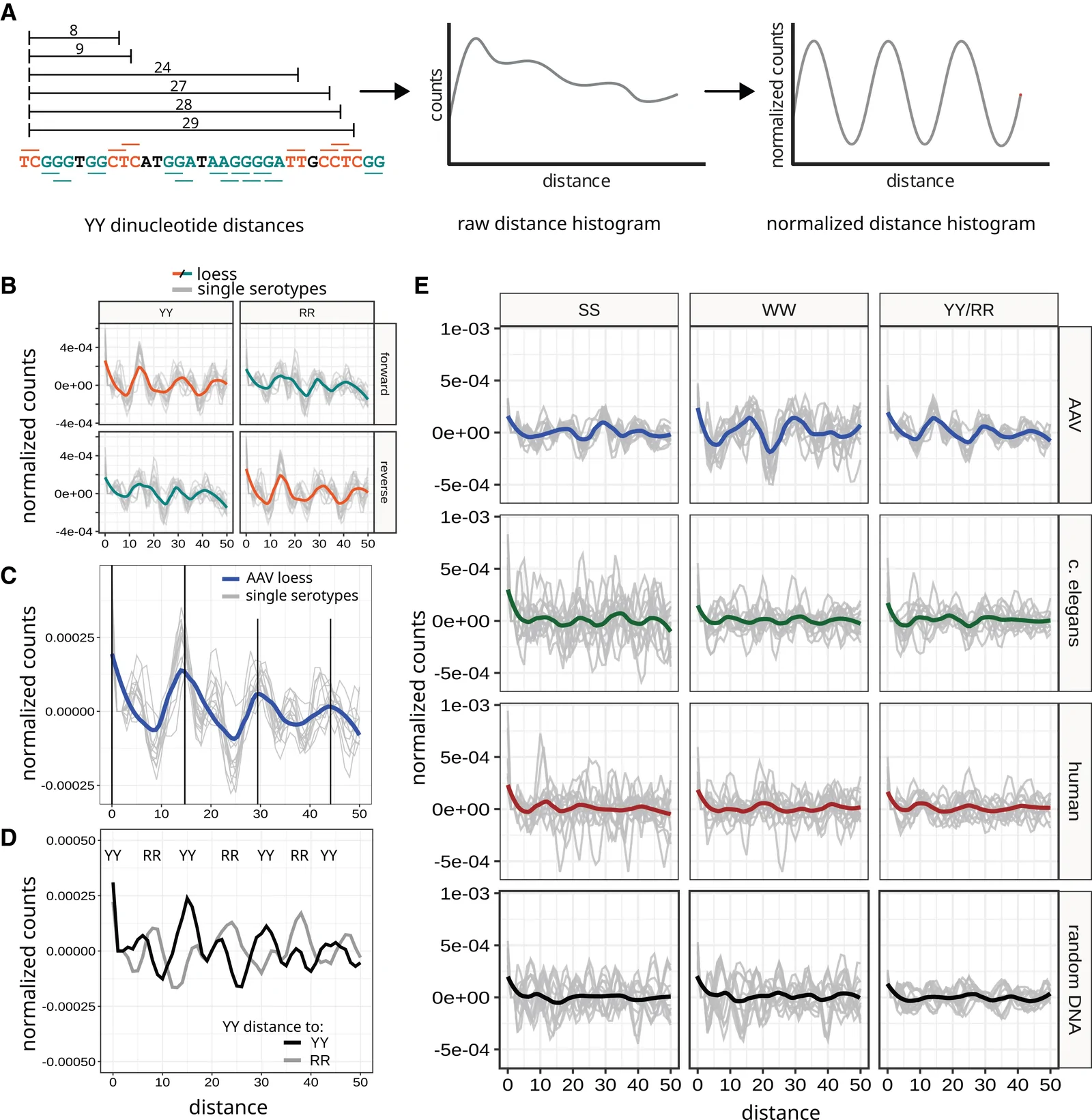
YY/RR periodicity in 13 primate AAV serotypes (A) Workflow of dinucleotide histogram calculation. The distance from each YY dinucleotide to every other (red) is recorded, exemplified for the first YY dinucleotide (TC in this example). RR dinucleotides (green) are YY on the opposing strand. The distance histograms are then smoothed and normalized in a final step. (B) Distance histograms for the YY and RR dinucleotides on both strands of all 13 primate AAV serotypes. Each gray line is the histogram of one serotype. The red line represents the loess of the YY pattern on the forward strand (RR in reverse). The green line represents the loess of the RR pattern on the forward strand (YY in reverse). (C) Combined distance histogram of YY and RR on both strands of the 13 primate AAV serotypes with equally spaced vertical lines with a period of ∼14.8 bp. (D) Distance histogram of YY dinucleotides to other YY and RR dinucleotides showing how a YY and an RR pattern can exist on the same strand by being shifted relative to each other. (E) Histograms for different dinucleotides in sequence sets from AAV, model organisms, and randomized DNA. As a control, 13 5-kb-long sequences were randomly selected from the coding regions of *C. elegans* and human or generated for random DNA. Each gray line represents a single sequence, and the colored lines represent the loess of all sequences. YY: CC, TT, CT, or TC. RR: GG, AA, GA, or AG. SS: GG, CC, CG, or GC. WW: AA, TT, AT, or TA.

Generally, the analysis of dinucleotide periodicity entails determining all positions of a specific dinucleotide, calculating a distance matrix among those coordinates, and then computing a histogram of these distances (Figure 1A). The counts of this histogram are subsequently processed in two additional steps: (1) a smoothing window of 3 bp is applied to minimize the omnipresent 3 bp periodicity and (2) a background window of 7 bp is subtracted from the histogram for normalization.^28^^,^^32^

We first interrogated the genomic sequences of the 13 known primate AAV serotypes for their periodicity. Since AAV is an ssDNA virus, we initially analyzed multiple dinucleotides in both strands separately for every individual viral genome sequence. The most significant pattern on both strands was observed for the YY dinucleotide combination (CC, CT, TC, and TT) and its reverse complement RR (Figure 1B). Both showed a periodicity in which the dinucleotides repeated every ∼15 bp when averaging the 13 AAV serotypes. Smaller peaks that are not present in all sequences were located between the 15-bp peaks with a periodicity of 7–8 bp. Other dinucleotides and combinations thereof showed either non-periodic patterns or patterns that were apparent on only one strand at a time (Figure S1). Since the YY and RR patterns appeared similarly on both strands (Figure 1C), and since YY/RR is always complementary to the opposing strand, we analyzed both strands simultaneously from here on. Attempts to further delineate a preferred localization of the pattern within the AAV genomes did not reveal any particular region with increased periodicity (data not shown), likely explained by the short size of the AAV genome.

Given that both a YY and an RR pattern exist on the same strand simultaneously, the two patterns must be shifted relative to each other. To analyze this, we assessed the distances between YY dinucleotides and not only other YY but also RR dinucleotides (Figure 1D). In other words, from the perspective of each YY pair, we recorded the distance to other YY but also to all RR dinucleotides. By doing this for the 13 aforementioned serotypes in a pooled approach, we observed that the RR pattern seems to be shifted by ∼8 bp relative to the YY pattern, which allows both 15-bp patterns to co-exist on the same strand (Figure 1D).

Despite the small individual sizes of AAV genomes, the YY/RR pattern seen on the 13 genomes (4.1–4.8 kb in size) appears remarkably pronounced. For comparison, we also included similar analyses of multiple dinucleotides in 13 random stretches (each 5 kb in size) from coding sequences of *C. elegans* and human and from randomized DNA (Figure 1E). Only in *C. elegans* can the 10-bp eukaryotic pattern be clearly discerned, notably, though, in the dinucleotides SS (GG, CC, GC, and CG) and WW (AA, TT, AT, and TA) as opposed to YY. In humans, in the SS and YY dinucleotide histograms, only the first peak at around 10 bp can be seen, after which the pattern begins to fade. The fact that the well-described eukaryotic pattern is not always visible in similarly short stretches of eukaryotic genomes underscores the clarity of the pattern in the analyzed AAV serotypes.

### The YY sequence pattern is unique for the genus *Dependoparvovirus*

**Figure 2:**
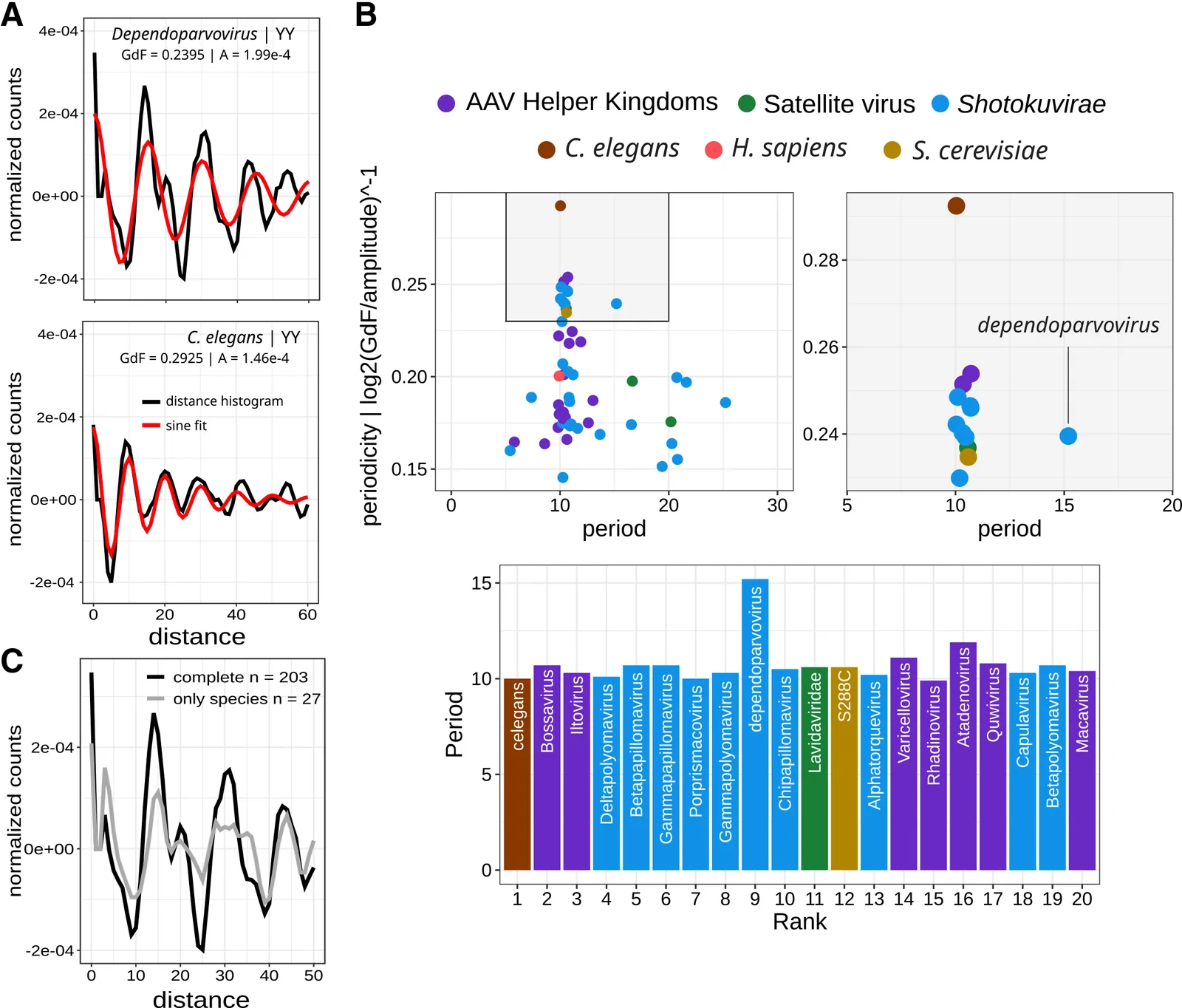
Periodicity quantification in selected viral genera (A) Normalized histograms of the YY/RR dinucleotide distances (black) and associated sinoid fits (red) for the genus *Dependoparvovirus* and the entire genome of *C. elegans*. (B) Relationship of YY periodicity and period of the fit for multiple sequence sets from genera of *Shotokuvirae*, satellite viruses, families from AAV helper viruses, and several control genomes. The top left depicts the total data, with the horizontal line representing the cutoff for the top hits defined at 0.23. The shaded rectangle is shown in more detail in the top right, with the *Dependoparvovirus* sequence set indicated. The bottom shows a bar plot of the period of the fit of the 20 most YY-periodic sequence sets ranked by periodicity. (C) Comparison of the normalized YY distance histogram between the complete *Dependoparvovirus* sequence set and a set containing only the 27 *Dependoparvovirus* reference species as listed by the ICTV.

To gain a deeper understanding of the prevalence of the YY/RR dinucleotide pattern, we expanded our analysis to the kingdom of *Shotokuvirae*, satellite viruses, AAV helper viruses, and control genome sequences. The *Shotokuvirae* kingdom encompasses mostly ssDNA as well as some double-stranded (ds)DNA viruses. Since AAV is an ssDNA virus and since the measured genomic pattern deviates from the well-described 10-bp repeat in eukaryotes, we started out by specifically analyzing other ssDNA viruses to ascertain whether the YY/RR pattern is a fundamental characteristic of their genome organization. Because of the small size of viral genomes, we opted to analyze all sequences from the same genus as one combined set of sequences. We took only genera with at least 100 kb of combined sequence length into consideration, which resulted in a total of 64 sequence sets comprising more than 25,000 unique sequences from 35 genera within the kingdom *Shotokuvirae*, as well as satellite viruses and AAV helper viruses. To aid in the comparison, we adapted a previously published quantification strategy that utilizes the fit of a damped sine function to model the periodicity of the distance histogram (Figure 2A).^28^ This was done for the YY, SS, and WW dinucleotides, of which the YY dinucleotide produced the most periodic hits (Figures 2B and S2). We observed that the majority of measured periods for all tested dinucleotides lies between 10 and 11 bp, which is especially evident at the top of the periodicity measurements (Figure 2B, top right panel). The genus *Dependoparvovirus* (containing all publicly available AAV sequences) ranks among the top hits for its YY periodicity. The full set of tested sequences and associated measured values is found in Table S1. Remarkably, the 15-bp period stands out and is detected neither in further genera of *Shotokuvirae* nor in other satellite viruses, making it a unique feature of the genus *Dependoparvovirus* (Figure 2B, bottom panel).

Because the majority of deposited *Dependoparvovirus* sequences were extracted from ducks, geese (avian/anseriform dependoparvoviruses), or primates (AAV), this sequence set might be biased toward viruses infecting those hosts. To interrogate the existence of such a bias, the normalized YY distance histogram of the entire set of 203 *Dependoparvovirus* sequences was compared with a set composed of the reference genomes of each of the 27 different *Dependoparvovirus* species as listed by the ICTV (International Committee on Taxonomy of Viruses).^33^ As seen in Figure 2C, the 15-bp YY periodicity is still clearly detectable in the smaller *Dependoparvovirus* set, albeit at a lower intensity reflected by peaks and valleys with fewer normalized counts.

### The 15-bp YY periodicity is governed by selection

The presence of this unique periodic pattern on AAV genomic DNA indicates that it likely provides an advantage for the virus. We therefore designed an experiment to assess whether sequences with higher periodicity will be selected under iterative pressure. Specifically, we applied DNA family shuffling technology to closely related AAV capsid (*cap*) sequences, yielding a library of millions of chimeric viruses that can be subjected to iterative selection for, e.g., better encapsidation of viral DNA or infection of target cells. In the gene therapy field, this method is frequently used to identify AAV capsid variants with antibody-evading abilities and other clinically relevant properties.^34^^,^^35^^,^^36^^,^^37^

**Figure 3:**
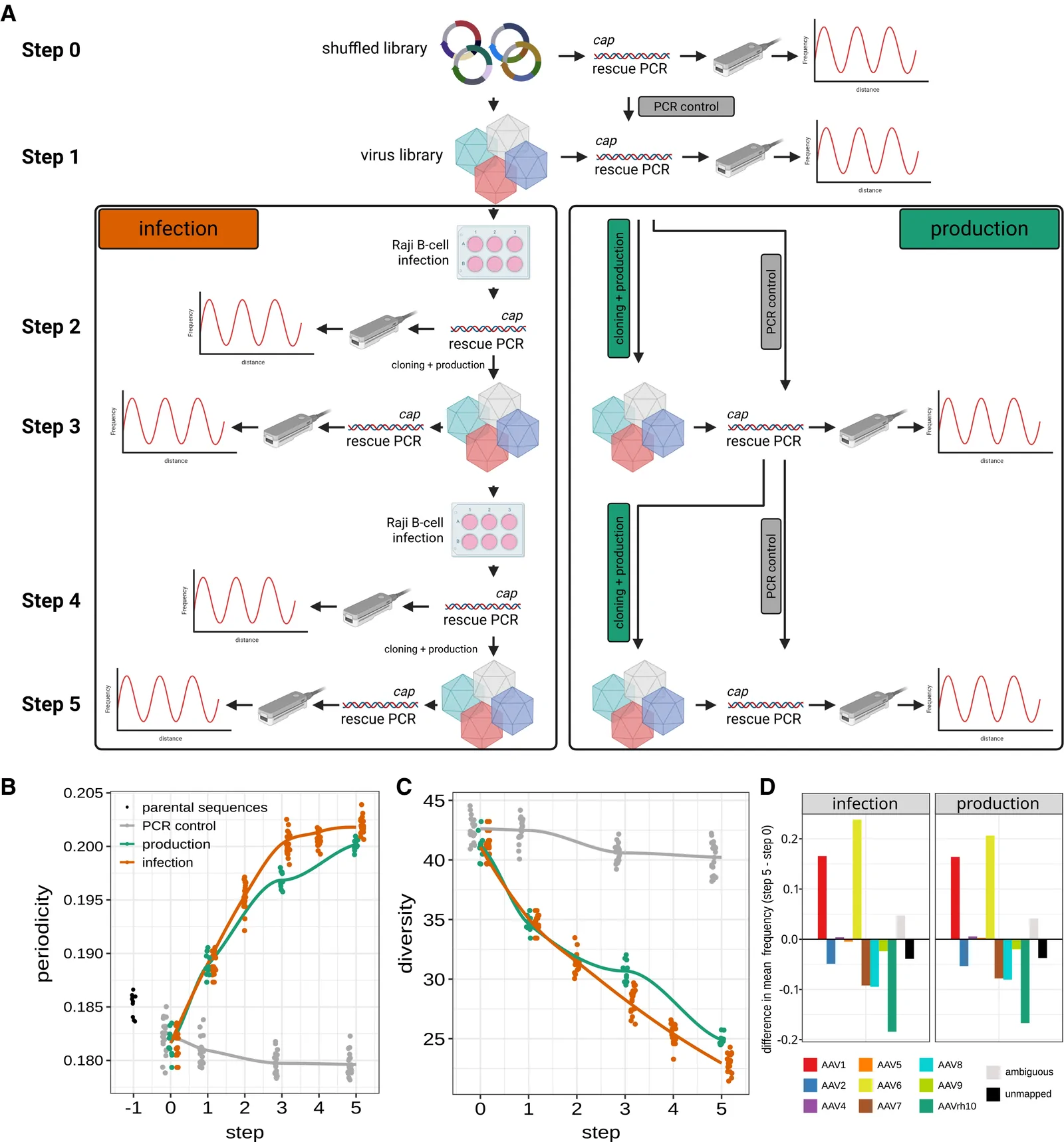
*cap* gene sequences become more periodic under selection (A) Illustration of the experimental setup. A DNA-family-shuffled *cap* gene library was used to produce viruses, which were either used to infect Raji cells and then amplified from the cells (infection) or directly amplified without an infection step (production). Next, each of the amplified *cap* libraries was used to produce a secondary virus library that was processed once as before. At every step, part of the *cap* amplicon was retained for Nanopore sequencing and subsequent quantification of periodicity. Next to the infection and production halves, a PCR control was included that consisted of repeated PCR amplifications of the initial shuffled *cap* gene library. (B) Increase in periodicity over the selection steps. Here, the parental sequences (AAV1 to AAV9 and AAVrh10) were also analyzed to simulate an additional step −1. Each dot represents a random subsample of 600 sequences from the sequenced *cap* genes and deviates slightly along the *x* axis for clarity. The line represents a loess along the individual groups of dots. (C) Loss of diversity along the selection steps. Every point represents the same subsamples of 600 *cap* genes as in (B). Here, diversity is a measure of how many clusters a cd-hit-est clustering approach produces at differing sequence similarity thresholds (see materials and methods). (D) Difference in mean parental frequency between step 5 and step 0.

Here, we used this procedure to partially disrupt the inherent periodic pattern on the viral DNA and then to analyze which chimeric sequences are enriched under a specific selection pressure. To this end, the parental *cap* genes of AAV serotypes 1 through 9 as well as of the variant rh10 were shuffled to generate an initial *cap* gene-encoding plasmid library. Next, these sequences were used to generate a virus library in which, ideally, every capsid packages the viral genome that encodes it. This virus library was either directly subjected to PCR amplification of the packaged *cap* genes, in order to enrich and study those sequences that excel at encapsidation, or, alternatively, used to infect cultured Raji cells (a human B lymphoblastoid cell line), followed by amplification of all *cap* variants that had successfully entered the cells (Figure 3A, production and infection sides, respectively). In both scenarios, the amplified *cap* sequences were then used to generate a secondary virus library for a second round of identical selection. Moreover, we included a control in which we amplified only the *cap* sequences without subjecting them to the selective pressures of infection or production.

At every step, Nanopore sequencing was performed to assess the periodicity of the amplified *cap* genes. The Nanopore reads were filtered by a minimum quality of 12 and an expected *cap* gene length of ∼2.2 kb, which left between 2.3 × 10^3^ and 8.7 × 10^3^ unique *cap* sequences per sample available for analysis (Figures S3A and S3B). To keep the computation time within a reasonable limit, we subsampled 600 reads from the total sequenced reads 10 times, enabling us to assess the periodicity as in Figure 2.

As intended, we found that the periodicity of the parental sequences was indeed disrupted by the DNA shuffling (Figure 3B, steps −1 to 0). Interestingly, over the course of selection, the periodicity of the capsid sequences continually increased for both the infection and the production arms of the experiment. In contrast, and as expected, it remained unchanged in the PCR controls. Throughout the entire selection procedure, the periodicity of the YY/RR pattern persisted at 15 bp (Figure S3C). Selected sequences were characterized by the deepening of the first valley at ∼9 bp and the appearance of a clearer second peak at ∼30 bp (Figure S3C, arrows), which likely led to the measured increase in periodicity.

To assess whether the observed sequences were truly selected, we additionally quantified the diversity of all sequences at every step in a sequence-clustering-based approach. The diversity of sequences recovered under both the production and the infection selection markedly decreased, whereas the PCR controls retained similar levels throughout all steps (Figures 3C and S3D).

Last, we employed a quick parental assignment workflow on the sequenced *cap* genes. Briefly, reads were split using a sliding window, and every window was aligned to a set of parental reference sequences (Figure S4A). Along the experimental steps, AAV serotypes 1 and 6 became more frequent, while others were depleted (Figures S4B and S4C). We summarized these results by calculating the mean parental content per read and comparing between steps 5 and 0 (Figure 3D). Importantly, no single serotype or sequence composition dominated the sequence pool.

Since we did not introduce any diversification beyond the initial *cap* library generation, these results reaffirm the notion that *cap* sequences are indeed selected based on their 15-bp YY/RR periodicity driven by production and infection pressures.

### Enhanced replication of DNA with YY periodicity

**Figure 4:**
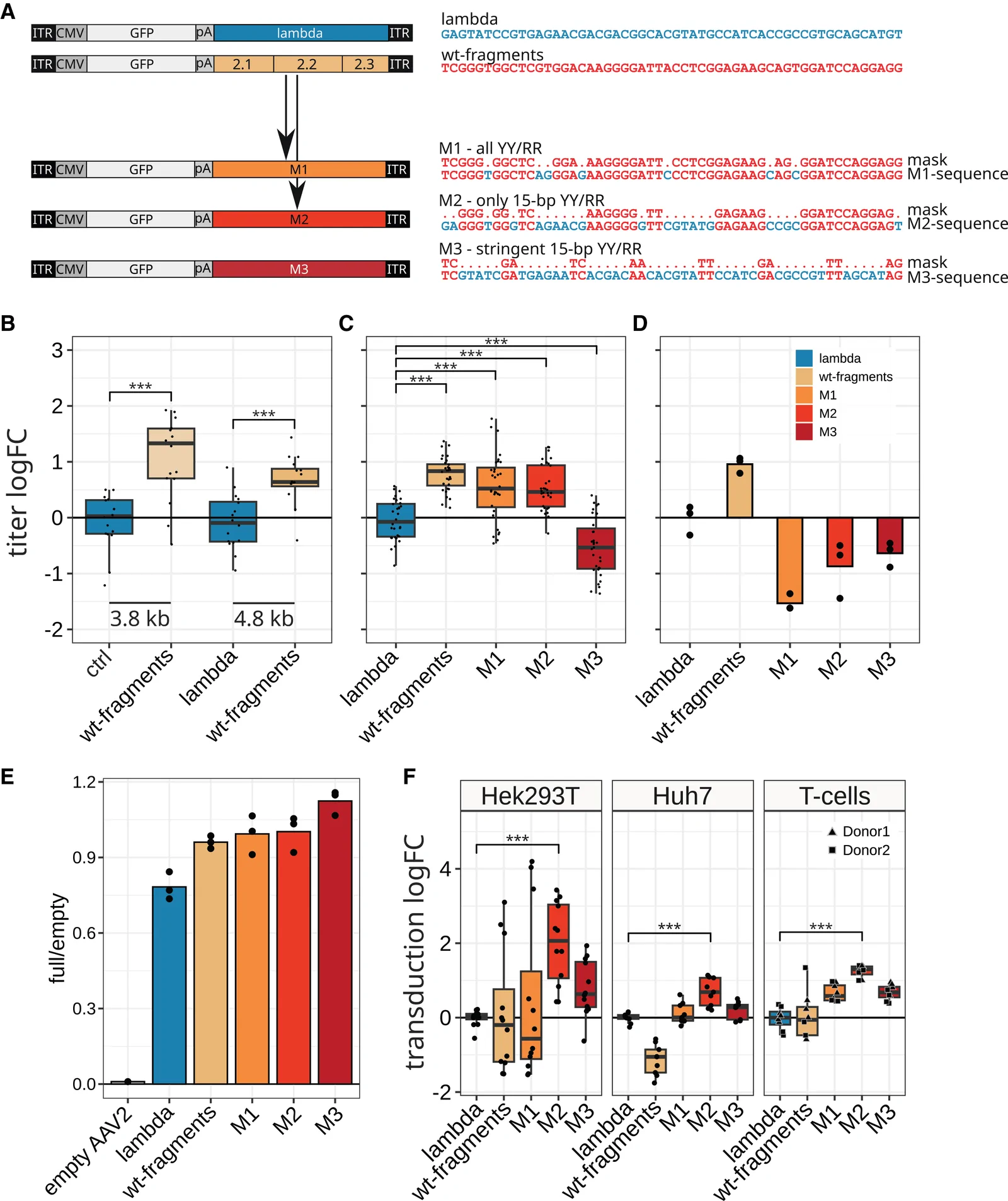
Insertion of periodicity into rAAV stuffer DNA affects viral titer and transduction All vectors were produced with AAV2 *cap*. (A) Generation of periodic transgene cassettes. The wt-fragments stuffer consists of three inert fragments of AAV2, all containing the YY periodicity. The lambda stuffer is not periodic. M1 copies all YY and RR dinucleotides of the wt-fragments onto the lambda DNA stuffer. M2 copies only YY and RR dinucleotides of the wt-fragments that are exactly 15 bp apart onto the lambda DNA stuffer. The M3 stuffer sequence contains a stringent YY/RR pattern regardless of the wt-fragments sequence. Depicted here are only the 4.8-kb-sized transgenes. (B–D) Nuclease-resistant virus genome titer represented as a log fold-change against the mean of the control. Wilcoxon test; ∗∗∗*p* < 0.001. (B) Small-scale virus production of control stuffer- and wt-fragments-containing cassettes with a size of either 3.8 or 4.8 kb. (C) Small-scale virus production of pattern-imitating cassettes M1 to M3 alongside the negative and positive controls. (D) Large-scale production of the same cassettes as in (C). Crude cell lysates were digested with benzonase and purified on an iodixanol gradient. (E) Ratio of full to empty particles measured by mass photometry following AAV affinity purification from cell lysates. Each dot represents a technical replicate, while the bar height represents the mean. (F) Transduction of periodic stuffer constructs in Hek293T, Huh7 and CD4^+^ T cells. T cells from two donors were transduced and are displayed in the same graph. Transduction is represented as a log fold-change against the vector containing the lambda stuffer. Wilcoxon test; *p* values are denoted by ∗∗∗ for *p* < 0.001.

To analyze the effect of the dinucleotide periodicity on the wild-type AAV genome in the context of an rAAV vector, we designed and tested different transgene cassettes in a small-scale production assay. To replicate the dinucleotide patterns found on the wild-type AAV genome as a positive control, we inserted fragments from the AAV2 *rep* and *cap* genes as stuffer DNA into a transgene cassette (wt-fragments). These transgenes do not contain the start or stop codons of any known AAV protein or known regulatory elements. These positive controls were created to be either 3.8 or 4.8 kb in size (Figure 4A, top; only the 4.8-kb variant is shown). As control stuffers, the sequences of either the prokaryotic kanamycin resistance gene (3.8 kb transgene; not shown) or lambda phage DNA (4.8 kb transgene; Figure 4A) were used. When produced in small scale, the wt-fragments transgenes show a 0.7 to 1.3 times log-fold increase in DNase-resistant particles compared with their controls, regardless of genome size (Figure 4B). This indicates that the wild-type sequence fragments contain a DNA-encoded signal influencing their production.

To assess whether the dinucleotide pattern in these sequence fragments plays a role in this increase, we designed three additional stuffer DNA sequences, namely, M1, M2, and M3 (Figure 4A, bottom). In these, we mapped the wt-fragments dinucleotide pattern onto the lambda control sequence using different masks. M1 uses a mask of all YY or RR dinucleotides that are present on the wt-fragments. Since this mutates 80% of the lambda sequence, we also designed M2 in which the mask contains only YY dinucleotides that are exactly 15 bp apart from another YY (and the same for RR), which mutates only slightly less than 50% of the lambda DNA. The mask for sequence M3 contains 15-bp perfectly spaced repeats of random YY and RR dinucleotides shifted by 8 bp to one another. Distance histograms of the YY and RR dinucleotide patterns showed the expected result (Figure S5A). While the M1 pattern stays close to that of the wt-fragments, M2 has an exaggerated peak at 15 bp, and M3 possesses a highly exaggerated and regular distance histogram. Based on the applied mask, M1 and M2 sequences are closely related to the wt-fragments sequence, whereas the M3 sequence is more similar to the lambda stuffer sequence (Figure S5B).

Next, we assessed the production capabilities of the designed cassettes. First, small-scale production in a 96-well plate revealed that M1 and M2 behave very similarly to wt-fragments, producing about 50% more DNase-resistant particles compared with the control (Figure 4C). Notably, M3 did not follow this trend and fell short of the control. Surprisingly, when the same transgene cassettes were packaged on a larger scale, including iodixanol gradient purification, only the wt-fragments cassette mirrored the pattern of the small-scale productions (Figure 4D).

As possible reasons for the differences in viral titers, we investigated genome integrity, full-to-empty particle ratios, and secondary DNA structure formation. To assess genome integrity, Nanopore sequencing was performed on vector productions, and long reads (>1 kb) were aligned to the reference transgene cassettes. All cassettes except for wt-fragments showed consistent coverage and low insertion/deletion rates (Figure S6). The wt-fragments cassette displayed a partial deletion of fragment 2.2 or 2.3 in about 42% of all reads. Similarly, a Southern blot analysis provided no evidence for aberrant replication intermediates but confirmed the presence of recombinations within the wt-cassette (Figure S5C). These are, however, unlikely to have affected the titer measurements as the latter are PCR based using a primer/probe set targeting only the CMV promoter in the viral genomes.

Moreover, we measured full-to-empty particle ratios in affinity-purified vector stocks using mass photometry. Particles were defined as full if their mass was between 5 and 6 MDa and as empty if it was between 3.5 and 4.5 MDa (Figure S7). Interestingly, the full-to-empty ratio was notably increased for all constructs compared with the lambda control (Figure 4E).

Secondary structure formation was predicted in a sliding window approach using the ssDNA parameters of ViennaRNA. This identified no areas within any stuffer DNA with a significantly decreased free ensemble energy (Figure S8).

Finally, we compared the constructs in an *in vitro* transduction assay. To this end, Hek293T or Huh7 cells were transduced at a multiplicity of infection (MOI) of 10^4^, and GFP emission was measured after 3 days. Similarly, primary human CD4^+^ T cells were extracted from leukopaks of two donors, activated for 2 days, and transduced at MOI of 10^5^ or 10^6^. GFP emission was measured by fluorescence-activated cell sorting (FACS) 3 days post-transduction. Intriguingly, the M2 cassette consistently yielded significantly greater GFP expression compared with the control in both cell lines as well as in the primary cells (Figure 4F). Even though the wt-fragments were better producers (see above), this was not reflected in the transduction experiments, as they were outperformed by the negative control (the vector with lambda DNA) in Huh7 cells.

## Discussion

Among all the known viruses, AAV is unique for both its fundamental biology that requires co-infection with an unrelated helper virus for AAV replication and its enormous promise as a recombinant vector for therapeutic gene transfer in humans. Over the past six decades, this has motivated a plethora of research studies aimed at dissecting the AAV life cycle and at concurrently unlocking the potential of AAV vectors via engineering of the viral genome, the encoded proteins, as well as the capsid structure and surface.^1^^,^^38^^,^^39^ In this study, we critically expand the ensuing knowledge with our discovery of a feature in wild-type AAV that has been understudied in the past, namely, a distinct and unique 15-bp YY/RR dinucleotide sequence pattern present in the genus *Dependoparvovirus* and, thus, in all AAV sequences.

While the exact mechanisms governing the functional relevance of this pattern remain elusive and a subject for future work, our data strongly imply that this pattern has emerged and been enriched over the course of natural virus evolution. One indirect evidence is our finding that periodic AAV genomes preferentially accumulate in virus particles during selection of a library of shuffled capsid genes (Figure 3), indicating an advantage for packaging, replication, and/or transduction. Additional support for the importance of the 15-bp YY/RR pattern comes from our observation that the inclusion of wild-type periodic DNA fragments in recombinant genomes positively impacts vector production, full-to-empty particle ratios, and transduction (Figure 4).

A tempting hypothesis is that this sequence pattern facilitates specific DNA-protein interactions that benefit the functionality of wild-type AAVs and rAAVs. The fact that we found this particular periodicity exclusively in *Dependoparvovirus* sequences suggests that its function is unique to a process inherent to this genus. One interesting candidate in this context is the adenoviral ssDNA binding protein (DBP) encoded by the E2A gene, which constitutes a seminal helper factor for rAAV production that binds about 12 nucleotides of ssDNA.^11^^,^^40^ However, the genus *Mastadenovirus* (adenoviruses) lacks a similar periodicity (Table S1), and the *Dependoparvovirus* genus also comprises viruses other than AAV that autonomously replicate without DBP, which together indicate that such an interaction is likely not universal. Moreover, the 15-bp YY/RR pattern does not represent a general feature of satellite viruses, as these are typically not very periodic overall or at least do not deviate from the common 10-bp periodicity (Table S1).

An alternative second explanation is that *Dependoparvovirus* Rep and VP proteins preferentially interact with their cognate viral DNA through this embedded pattern. Indeed, interactions between the DNA and the lumen of the capsid were described for other parvoviruses^41^^,^^42^^,^^43^^,^^44^ and also for multiple AAV serotypes.^45^^,^^46^^,^^47^^,^^48^ In AAV, the DNA-binding pockets of the capsid were reported to be accompanied by nucleotide densities attributable to packaged DNA, yet a connection to function was never established. In addition, only a single nucleotide or dinucleotide (A or AC) was resolved in the binding pocket, suggesting a relatively minor association of the viral DNA with the capsid. Because the binding pocket is also occupied in rAAVs with a non-wild-type genome, it was concluded that this binding is likely not sequence specific.^47^^,^^48^ Notably, this conclusion was drawn assuming that DNA-capsid interaction requires a distinct binding motif without considering the propensity of patterned DNA to mediate this interaction, too. In fact, an increased interaction of the periodic wild-type AAV genome with the capsid could readily explain the better packaging of the wt-fragments cassette in small- and large-scale production (Figures 4B–4D), making it an interesting topic for specific further investigation in follow-up work.

Third, a key role of this pattern could also relate to the helicase activity of Rep, which is necessary for AAV genome replication and encapsidation. AAV Rep is classified as an SF3 helicase but additionally possesses uncommon properties, such as an unconventional oligomerization behavior.^49^^,^^50^^,^^51^ In recent cryoelectron microscopy studies, the heptameric complex of Rep68 proteins bound to a dsDNA substrate was shown to encompass roughly 21 bp, of which 15 bp had clearly defined densities.^49^ Their final model of the bound DNA had a length of 14 bp of clearly resolved DNA, suggesting that a Rep68 heptamer binds tightly to 14 bp of dsDNA. This is remarkably close to the repeat length of 15 bp of the periodic AAV genome pattern, implying that the periodicity of *Dependoparvovirus* sequences may be critical for the binding of the viral DNA to Rep68 heptamers. The large Rep proteins Rep68 and Rep78 are necessary for AAV replication, as they melt dsDNA and catalyze the terminal resolution within the ITR sequences.^52^ Therefore, an increased affinity of Rep to viral DNA substrates may impact the formation of replication intermediates or replication efficiency, which could explain the higher titers of the periodic constructs M1 and M2 in small-scale production.

Last, even though we did not observe increased secondary structure formation in our designed transgene cassettes (Figure S8), it is conceivable that dinucleotide patterns aid in specific contortions of DNA, including AAV genomes. In the future, this could be investigated using molecular dynamics simulations of wild-type and recombinant ssDNA AAV genomes. If confirmed, this would then even further extend our knowledge of AAV biology and concurrently outline options for rational genome engineering with the aim to improve the packaging process and hence vector titers.

Regardless of mechanism, the data reported here have yet another implication, as they suggest the future use of the periodic pattern as a marker for *Dependoparvovirus* sequences, given that this pattern is distinct from the prevalent 10-bp periodicity present in eukaryotic and viral sequences. This is encouraging considering how the discovery of novel virus sequences has been revolutionized before by metagenomic high-throughput sequencing approaches and the subsequent assembly of viral genomes.^53^ Pre-selecting metagenomic datasets for their periodicity could thus benefit and accelerate the search for sets that are most likely to yield dependoparvoviral assemblies, similar to how the 3-bp periodicity is used in gene prediction.^22^ Thereby, this new approach could streamline and speed up the discovery of previously unknown AAV sequences, which, as recently demonstrated by us,^54^ can represent exciting templates for subsequent vector generation or further diversification through, e.g., DNA family shuffling.^36^^,^^55^^,^^56^

Until then, our work is the latest entry in a long list of research aimed at elucidating the factors and mechanisms that make wild-type, but not necessarily recombinant, AAV highly competent at production and transduction.^8^^,^^14^ To date, efforts to improve these parameters for AAV vectors have predominantly focused on capsid, ITR, Rep, or promoter engineering and screening^38^^,^^57^^,^^58^^,^^59^^,^^60^; the inclusion of cryptic packaging elements beyond the ITR, such as CARE^61^^,^^62^; and/or the improvement and upscaling of AAV vector manufacturing protocols.^63^ Through the discovery of a periodic sequence pattern in wild-type AAV DNA that is inadvertently lost during the generation of rAAV genomes, our study adds another parameter that has great potential to ultimately enable the creation of AAV vectors with near-wild-type efficiencies and thereby aid in realizing the promise of AAV-mediated human gene therapy.

## Materials and methods

### Distance histogram calculation

The method to calculate the distribution of distances from a dinucleotide to every one of its occurrences was adapted from the Bioconductor package “periodicDNA.”^64^ Briefly, the location of every dinucleotide is recorded in a list on which a distance matrix is calculated. To reduce working memory requirements, first, sequences with a length of above 10,000 bp were split into subsequences with a maximum length of 10,000 bp, and second, only distances below 300 bp were taken into consideration. Subsequently, a histogram of the resulting distances is calculated. The counts in the histogram are processed in two steps. First, they are smoothed by averaging over a 3-bp window to reduce the influence of the 3-bp pattern in coding sequences. Then, the background level is calculated by averaging over a 7-bp window and subsequently subtracted from the smoothed histogram. This procedure results in the normalized counts of distance histograms in all figures. The background subtraction leads to the first 3 bp not being normalized and remaining abnormally high. Therefore, in the plots, these 3 bp were set to 0. Similarly, to simulate that position 0 contains the measured dinucleotide, that position was set to 1.3× the value of the maximum in the first 50 bp of normalized counts. For visualizing the convergence of multiple such distance histograms, a ggplot2 “geom_smooth” object with “method=loess” and “span=0.3” was used (Figures 1 and S1).^65^^,^^66^

### Quantifying goodness of fit and periodicity

To quantify the periodicity of a set of sequences, the goodness of fit (GdF) was calculated to a damped sine wave as described.^28^ Briefly, a sine wave was fit as described in Equation 1 to the normalized counts distance histograms, where H is the half-life, λ the period, and A the amplitude of the fit:

(Equation 1) $$f\left(x\right)=e^{\frac{-\ln\left(2\right)x}{H}}*A*\sin\left(\frac{2\pi x}{\lambda }+\frac{\pi }{2}\right)\text{.}$$

The “nls” function of R with the Gauss-Newton algorithm, with a step size of 1/2,048 and a maximum of 10,000 iterations, was used to perform the fit. Initiation values for every fit were chosen to be A = 1 × 10^−3^, H = 20, and λ = 20. If the initial fit failed, initiation values were randomized 300 times in a specific range (A ∈ [5 × 10^−4^, 1 × 10^−2^], H ∈ {20..50}, λ ∈ {5..30}). GdF is here defined as the deviance of the fit divided by the standard deviation of the normalized histogram data (σ), as described in Equation 2:

(Equation 2) $$GdF=\frac{deviance}{\sigma }\text{.}$$

The fit with the lowest GdF value is considered the best GdF and retained. The strength of the periodicity (merely called periodicity in the results) is here defined as the reciprocal of the log of GdF over the amplitude A of the sine fit, as defined in Equation 3:

(Equation 3) $$periodicity=\log_{2}{\left(\frac{GdF}{A}\right)}^{-1}\text{.}$$

### Data gathering and periodicity calculations

Sequences for every genus were downloaded using the R package “rentrez,”^67^ with the query “*genus*[Organism] and ‘complete genome’.” Sequences below 1,000 bp were discarded, and sequence duplicates were removed with seqkit.^68^ This was done with all genera within the *Shotokuvirae* kingdom as listed in the ICTV. Genera with less than 1 million bp of total unique sequence information were omitted from the analysis. In addition to *Shotokuvirae*, the genera in the families *Adenoviridae* and *Orthoherpesviridae* were analyzed, since the most researched AAV helper viruses (adeno- and herpesvirus) belong to those families. Since AAV is a satellite virus, hepatitis D virus sequences and sequences associated with the terms *Lavidaviridae* and *Alphasatellitidae* were additionally analyzed. Since viral genomes predominantly consist of coding sequences, as a control, the consensus coding sequences of the human genome were downloaded from the Consensus CoDing Sequence project (https://ftp.ncbi.nlm.nih.gov/pub/CCDS/current_human/) and processed as above. Similarly, all ORF (open reading frame) sequences (orf_genomic_all) were downloaded for the *S. cerevisiae* S288C R64 assembly from the sgd archive (http://sgd-archive.yeastgenome.org/sequence/S288C_reference/orf_dna/). All coding sequences for *C. elegans* assembly WBcel235 were downloaded from ensembl (https://ftp.ensembl.org/pub/release-113/fasta/caenorhabditis_elegans/cds/). The *E. coli* sequence was obtained from the accession number GenBank: U00096.3.

To keep computation time to a minimum and also to keep individual genera comparable to one another, the individual sequence sets from every genus were subsampled to achieve a total length of 1 × 10^5^ bp. Subsampling was performed 10 times, and results were individually subjected to periodicity quantification as described above, before the subsample calculations were recombined to obtain the final values for any given genus. The period and GdF values are the median values from the 10 subsamples.

For the bias interrogation of the *Dependoparvovirus* sequence set, the archetypal sequences of the 27 *Dependoparvovirus* species as listed by the ICTV were analyzed.^33^ The same analysis as described above was performed, without subsampling the dataset. The individual accession numbers are GenBank: U22967, GenBank: MW046577, GenBank: MT138326, GenBank: KX583629, GenBank: MT138328, GenBank: AY186198, GenBank: MN175614, GenBank: MN794870, GenBank: OQ198157, GenBank: OQ198130, GenBank: OQ198151, GenBank: OQ198126, GenBank: OQ198128, GenBank: GU226971, GenBank: MG745677, GenBank: AF085716, GenBank: MT138301, GenBank: MT138277, GenBank: JN420372, GenBank: AF043303, GenBank: OQ101836, GenBank: MF416383, GenBank: MF416384, GenBank: MN242366, GenBank: MN242367, GenBank: AY349010, and GenBank: KP733794.

### DNA family shuffling and library selection

The capsid library consisted of the AAV capsid serotypes 1 through 9 and serotype rh10. The plasmid library consisted of an ITR-flanked AAV2 *rep* and a shuffled *cap*. Virus was produced in a dual transfection approach with an adenoviral helper plasmid (AdH) at a molar ratio of 1:20 (library:AdH), purified by iodixanol gradient, and subsequently size-filtered. For the infection of Raji B cells, 2 × 10^5^ cells in 2 mL RPMI 1640 medium (Gibco/Thermo Fisher Scientific, Waltham, MA, USA) were seeded in a six-well plate. Wells were infected with the virus library at an MOI of 1 × 10^5^ particles per cell. Cells were incubated for 2 days and then harvested by centrifugation at 300*g* for 5 min. The pellet was washed twice in PBS (phosphate-buffered saline; Gibco/Thermo Fisher Scientific, Waltham, MA, USA). To remove any AAV capsids sticking to the cell membranes, the pellet was incubated with 200 μL of 0.25% trypsin (Gibco/Thermo Fisher Scientific, Waltham, MA, USA) for 10 min at 37°C before it was washed twice in PBS as before. DNA was extracted from the cell pellet with the DNeasy Blood and Tissue Kit (69504, Qiagen, Hilden, Germany) according to the manufacturer’s protocol, which was used in the past to good effect.^35^ PCRs to rescue viral DNA directly from virus stocks or from cell pellets were performed with the Phusion Flash High-Fidelity PCR Master Mix (F548, Thermo Scientific, Waltham, MA, USA), with a final primer concentration of 400 nM, 1 μL template, and final volume of 50 μL in water. The resulting PCR amplicon was either processed by ONT (Oxford Nanopore Technologies) library preparation or recloned for another virus library generation step. For the latter, 3 μg of PCR amplicon was digested with AscI and PacI (NEB, Ipswich, MA, USA) overnight at 37°C and subsequently ligated with 500 ng of a similarly digested acceptor plasmid. The mix was ligated with T4 ligase (NEB, Ipswich, MA, USA) and incubated at room temperature overnight. The plasmid was transformed into electrocompetent cells by electroporation. Up to 11 electroporations were performed per ligation mix and mixed again afterward. Electroporation was performed by adding 1 μL of ligation mix to 30 μL of *E. cloni* 10G Supreme cells (Lucigen, Middleton, WI, USA) and pulsing on a Gene Pulser Xcell at 1.8 kV. Directly after electroporation, 970 μL SOB medium was added, and the bacteria were incubated at 37°C for 1 h. A sample of all electroporations of one ligation reaction was diluted 1:100,000 and plated on an LB-ampicillin plate. Library diversity was estimated by counting colonies that grew overnight.

### Nanopore amplicon sequencing

Nanopore libraries were generated using the Ligation Sequencing Kit from ONT (SQK-NBD114.24, Oxford, UK). The associated protocol (15 Sep 2022) was performed with 200 fmol of PCR amplicons as input per sample. Sequencing was performed on an Mk1B with an R10.4.1 flow cell. Reads were rebase called using Dorado^69^ with the latest SUP model and a quality threshold of 8. All reads were subsequently filtered by length (2,250 < read_length < 2,340) and Phred quality (read_quality > 12), which left between 23,000 and 87,000 reads per sample. The total reads were subsampled down to 600 reads 10 times using “seqkit sample -n 600” and exact sequence duplicates were removed using “seqkit rmdup -s” (0 duplicates in most cases). The 10 subsamples were assessed for their YY/RR periodicity in the same way as described above.

### Nanopore AAV sequencing

Vector was produced in large scale as described below. After iodixanol purification, buffer was exchanged to DPBS using an Amicon Ultra-15 Centrifugal Filter Unit (MWCO 100,000; Merck, Darmstadt, Germany). Library preparation was performed according to the ONT protocol “AAV_9194_v114_revJ_07Oct2025” using the PureLink Viral RNA/DNA Mini Kit (Thermo Fisher, Waltham, MA, USA; 12280050) and the Native Barcoding Kit 24 V14 (ONT, Oxford, UK; SQK-NBD114.24). Genome integrity analysis was performed by aligning long reads from the sequencing (>1 kb) to the transgene reference genomes using minimap2,^70^ from which only primary alignments were retained. Pileups from the alignment were created and visualized in R using ggplot2^65^ and Rsamtools (https://doi.org/10.18129/b9.bioc.rsamtools). Coverage was extracted using the GenomicAlignments package.^71^

### Diversity analysis

The clustering program cd-hit-est was used to measure the diversity of the *cap* sequences from the shuffling enrichment experiment.^72^ The 600 sequences from every subsample were clustered with cd-hit-est in the accurate mode while comparing both strands (parameters “-r 1 -g 0 -sc 1”). This was done while iterating over the sequence similarity threshold, which dictates cluster formation. For every iteration, the number of resulting clusters was retained and normalized to the number of sequences. The area under the resulting curve was estimated by summing up the percentages over all sequence similarity thresholds, which is here called diversity. The curves are shown in Figure S3D.

### Parental assignment

We employed a method for quick parental assignment that was inspired by hafoe.^73^ In short, we employed a sliding window approach with window size 100 bp and step size 10 bp on each ONT-obtained *cap* sequence and aligned every window individually to all parental *cap* sequences (Figure S4A). Similar to the diversity analysis, the set of query sequences consisted of 500 randomly chosen sequences with the same filtering steps as described before (2,250 < read_length < 2,340 and 12 < mean_phred_quality). Alignment was performed using minimap2 with the command “minimap2 -c -x map-ont -k 10 -w 5”. Windows were counted as ambiguous if the mapping quality of all matches for a specific window were equal to 0 or if multiple hits did not differ by more than 5 in mapping quality. For visualization, the ratio of every parental sequence within every sample *cap* read was calculated.

### Cloning and plasmids

All plasmids were generated by restriction cloning. The fragments for M1, M2, and M3 were designed *in silico* and synthesized by Twist Bioscience (South San Francisco, CA, USA) as gene fragments. All plasmid sequences were confirmed by whole-plasmid sequencing.

### Small-scale production

Production in 96-well plates was performed using Hek293T cells seeded in DMEM substituted with 10% FBS and 1% penicillin-streptomycin (Gibco/Thermo Fisher Scientific, Waltham, MA, USA). Cells were seeded in the 96-well plate at a density of 1.25 × 10^4^ cells in 100 μL medium per well and incubated overnight. To ensure an equal amount of plasmid DNA in every well, before transfection, plasmid DNA was quantified using a Qubit fluorometer. Using PEI-MAX (Polyscience Europe, Hirschberg an der Bergstrasse, Germany), each well was transfected with 6 fmol each of adenoviral helper plasmid, rep2/cap2 helper plasmid, and ITR-flanked transgene plasmid. To maintain minimal deviation in the amount of added helper plasmids, a master mix of adenoviral helper and rep2/cap2 plasmid was prepared (per well: 1.633 μL 300 mM NaCl, 6 fmol rep2/cap2, and 6 fmol adenoviral helper). Similarly, a PEI master mix was prepared (per well: 1.633 μL 300 mM NaCl, 0.9 μL nuclease-free water, and 0.73 μL PEI-max). To account for pipetting errors, every column of eight wells of the 96-well plates was transfected with an individually prepared transfection mix. One transfection mix (enough for 12 wells) contained 20.4 μL of helper mix and 72 fmol of transgene plasmid topped with water to a total volume of 39 μL. The transfection was finalized by adding 39 μL of PEI master mix, vortexing, and incubating for 10 min at room temperature. Each well was transfected with 6.5 μL of transfection mix, and the plate was slightly agitated to mix, followed by a 72-h incubation at 37°C.

Cells were dislodged by pipetting the medium and transferred to a new Eppendorf tube. The plate was then washed with 50 μL DPBS, which was also added to the same cup. The harvested cells were subjected to five rounds of freeze-thaw cycles and subsequently centrifuged for 10 min at 1,000*g*. Five microliters of the cell lysate was mixed with 37 μL water, 5 μL DNase I buffer, and 3 μL DNase I (2,000 U/mL, NEB M0303L, Ipswich, MA, USA); mixed by pipetting; and incubated at 37°C for 15 h. To inactivate the DNase, first, 6 μL of 50 mM EDTA was added to each cup and mixed by pipetting; second, the reaction was incubated at 75°C for 15 min. The DNase digestion was then diluted 1:1,000 in nuclease-free water and quantified via ddPCR with primer/probe sets against the CMV enhancer (see below for sequences) and β-lactamase (bla_fwd, GCATCTTACGGATGGCATGA; bla_rev, GTCCTCCGATCGTTGTCAGAA; bla_probe, HEX-CAGTGCTGCCATAACCATGAGTGA-3IABkFQ). The β-lactamase concentration was measured to gather information on the remaining plasmid contamination after DNase digestion. All transfected plasmids contained a β-lactamase gene, but only one out of three also contained the CMV enhancer of the transgene. Therefore, the CMV enhancer concentration was subtracted by one-third of the calculated β-lactamase concentration, resulting in the plasmid-corrected CMV enhancer values. The titers of the control transgene (Kana or lambda) were averaged, and all titers are displayed as log2 fold-change values compared with that average.

### Large-scale AAV production and purification

For large-scale AAV production, Hek293T cells were seeded on 15-cm cell culture plates (4 × 10^6^ cells in 22 mL complete medium per dish) using two plates per replicate. Two days later, the cells were triple-transfected with equimolar ratios of the AAV transgene, AAV2 helper, and adenoviral helper plasmids using PEI-MAX and a total of 25 μg DNA per plate. After 3 days, the cells were lifted, pelleted by centrifugation at 800*g* for 15 min, and resuspended in 5 mL benzonase buffer (150 mM NaCl, 50 mM Tris-HCl [pH 8.0], 2 mM MgCl_2_) per replicate. Cell lysis was achieved by five consecutive freeze-thaw cycles of incubating in liquid nitrogen and a 37°C water bath. Lysates were then cleared by two centrifugation rounds of 10 min at 4,000*g* to remove cell debris (pellet). AAVs were then purified through iodixanol gradient density centrifugation as described.^74^ Briefly, cleared lysates were loaded onto reseal polyallomer centrifuge tubes (16 × 76 mm; Seton Scientific, Petaluma, CA, USA) and underlaid with 2 mL each of 60%, 40%, 25% and 15% iodixanol phases (OptiPrep Progen, Heidelberg, Germany). After ultracentrifugation at 50,000 rpm for 2 h at 4°C (Optima L-90K ultracentrifuge and 70.1 Ti rotor, Beckman Coulter, Brea, CA, USA), the bottom-most 1.5 mL was discarded, and the following 0.8 mL (interphase between 60% and 40% iodixanol containing full AAV capsids) was collected. AAVs were titrated using ddPCR, with each reaction mix containing 10 μL of the ddPCR Supermix for Probes (no dUTP; Bio-Rad, Hercules, CA, USA), 3 μL nuclease-free water, 1 μL CMVenh (transgene) primer/probe mix, and 1 μL ITR-primer/probe mix (final concentrations: 900 nM for primers, 250 nM for probes; CMVenh_f, AACGCCAATAGGGACTTTCC; CMVenh_r, GGGCGTACTTGGCATATGAT; CMVenh_probe, FAM-CGGTAAACTGCCCACTTGGCAGT-BHQ1; ITR_f, GGAACCCCTAGTGATGGAGTT; ITR_r, CGGCCTCAGTGAGCGA; ITR_probe, HEX-CACTCCCTCTCTGCGCGCTCG-BHQ1) and 5 μL AAV template diluted 1:10^6^. The measured number of droplets positive for both transgene (CMVenh) and ITR was then corrected by the dilution factor and input volume to calculate the number of AAV particles per microliter.

### Transduction of Hek293T and Huh7 cells with fluorescence readout

Hek293T and Huh7 cells were seeded in 100 μL complete medium at a density of 1.5 × 10^6^ cells per well in 96-well flat clear-bottom, black-wall polystyrene microplates (Corning). Cells were immediately transduced with AAVs packaging wt-fragments, lambda control, or M1–M3 fragments at an MOI of 10^4^ vector genomes per cell (three wells per replicate, with three replicate large-scale AAV productions per construct). Three days after transduction, the supernatant was replaced with 100 μL PBS. For each well, eGFP fluorescence was quantified using a Spark Multimode microplate reader (Tecan, Männedorf, Switzerland).

### T cell isolation, transduction, and FACS

Human PBMCs were isolated from leukopaks (DRK Blutspendedienst, Mannheim, Germany) by density gradient centrifugation. CD4^+^ T cells were enriched using the EasySep Human CD4^+^ T cell Enrichment Kit (StemCell, Cologne, Germany), activated with CD3/CD28 Dynabeads (Thermo Fisher Scientific, Waltham, MA, USA; 25 μL per 10^6^ cells) in RPMI growth medium supplemented with 20 U/mL IL-2 (Peprotech; Thermo Fisher Scientific, Waltham, MA, USA), and 50,000 cells were plated in 96-well U-bottom plates for transduction after bead removal at 48 h. Transduction was performed at MOI of 1 × 10^5^ or 1×10^6^ for 20 h. Medium was exchanged and the cells were cultured for an additional 48 h. GFP expression was measured on a BD FACSCelesta flow cytometer (see Figure S9 for the gating strategy). Transduction rates were between 1% and 3% of the singlet cell population.

### Affinity purification

Each 5-mL freeze-thaw lysate (see above, large-scale AAV production) was pre-filtered through a 0.8-μm filter (Roth, Karlsruhe, Germany; P820.1) and subsequently passed through a 0.2-μm filter (Merck, Darmstadt, Germany; SLGVR33RB). Both filters were rinsed before and after sample loading with the equilibration buffer used for the downstream chromatography step (50 mM Tris-HCl, 150 mM NaCl, 2 mM MgCl_2_ [pH 8.5]). The filtered lysate was then loaded onto an ÄKTA Start chromatography system and purified by immunoaffinity capture using a POROS AAVX column (Thermo Fisher Scientific, Waltham, MA, USA; A36652), with elution performed at pH 3. The eluate was immediately neutralized by 1:10 dilution into 1 M Tris-HCl (pH 8.0). Subsequently, the sample was concentrated to a final volume of 250 μL using an Amicon centrifugal filter unit in PBS buffer (50 kDa molecular weight cutoff; Merck, Darmstadt, Germany; UCF8050). The final solution was sterile-filtered through a 0.1-μm filter (VWR, Darmstadt, Germany; 514-4130).

### Mass photometry

Mass photometry measurements were carried out using the TwoMP Auto instrument (Refeyn Ltd, Oxford, UK), 24-well sample gaskets (Refeyn Ltd, Oxford, UK; MP-CON-31004), and glass slides (Refeyn Ltd, Oxford, UK; MP-CON-21009). Calibration was performed at the start of the measurement run using empty AAV2 vectors diluted at a final concentration of 1 × 10^12^ capsids/mL. The empty AAV2 vectors were produced as described previously,^75^ collected from the first eluted fraction of the two-step anion-exchange elution, and characterized for concentration by ddPCR, for purity by silver staining, and for empty-to-full ratio by mass photometry. For each measurement, 5 μL of buffer (PBS) was added to the gasket and “droplet dilution” was selected to record the blank. Subsequently, 5 μL of sample at approximately 1 × 10^12^ particles/mL was mixed with the buffer droplet by pipetting up and down, and data acquisition was initiated with the “record” function. Each sample was recorded for 60 s using AcquireMP v.2024 R2.1 (Refeyn Ltd, Oxford, UK), capturing the binding and unbinding events. Each sample was measured in triplicate. AAV2 empty capsid mass calibration was performed in DiscoverMP v.2024 R2.1 (Refeyn Ltd, Oxford, UK) using the “Create a new calibration” option, based on a contrast value of −0.10650 obtained from the AAV2 empty capsid measurement and converted to a theoretical mass of 3,737.17 kDa. This mass calibration was applied to each sample. Raw data of events was exported and analyzed in R. Raw events were filtered for fit_error <0.1 and diffblur_error <23. Empty capsids were defined as calibrated values between 3.5 and 4.5 MDa. Filled capsids were defined as calibrated values between 5 and 6 MDa.

### DNA folding

ViennaRNA was used for secondary structure prediction of DNA segments.^76^ Similar to the parental assignment, the DNA sequences were split using a sliding window approach with a window size of 500 bp and a step size of 50 bp. For each 500-bp window, the forward and reverse sequences were predicted independently with the following command: “RNAfold --noPS --noDP -P DNA --noconv -p”. As a control, each window sequence was shuffled 100 times independently using ushuffle and a let size of 2 to preserve dinucleotide composition.^77^ For each prediction, the ensemble free energy was extracted and a *Z* score was calculated as described by Equation 4, where X_i_ is the free ensemble energy of window i, and μ_i_^s^ and σ_i_^s^ are the mean and standard deviation of free ensemble energies of the shuffled (s) control window i, respectively:

(Equation 4) $$z_{i}=\frac{X_{i}-\mu_{i}^{s}}{\sigma_{i}^{s}}\text{.}$$

### Statistical analysis

Groups were compared using the Wilcoxon rank-sum test (also known as Mann-Whitney U test) wherever denoted in the figure legends. A non-parametric test was used because a normal distribution could not be assumed for the data, which was also confirmed using the Shapiro test. Significance levels were as follows: ∗*p* < 0.05, ∗∗*p* < 0.01, and ∗∗∗*p* < 0.001.

## Data and code availability

Scripts for performing the reported analyses can be found in the GitHub repository https://github.com/ConradinBaumgartl/AAVperiodicity or on Zenodo (https://doi.org/10.5281/zenodo.18328074). Sequencing data from the Nanopore sequencing experiments can be obtained from the ENA with the project accession PRJEB106523.

## Acknowledgments

We are grateful to Gernot Längst for his exploratory experiments and helpful discussions. We thank Anne-Kathrin Kleider for the generation of the shuffled library used in this work. We are indebted to Frauke Mücksch and Jannik Löhr for supplying the CD4^+^ T cells used in this work. This work was supported by the 10.13039/501100010767Innovative Medicines Initiative 2 Joint Undertaking (JU) under grant agreement no. 945473 (ARDAT, to F.B. and D.G.). The authors are further grateful for support for J.B. and D.G. from the ANR-DFG-funded project MATRIXNASH.

## Author contributions

C.B.: conceptualization, data curation, formal analysis, investigation, methodology, software, supervision, visualization, writing – original draft, writing – review and editing. J.B.: conceptualization, investigation, methodology, visualization. L.N.: investigation, supervision. M.X.: investigation, methodology, supervision. E.G.: investigation. J.G.G.-C.: investigation. J.S.: investigation. S.H.: investigation. F.B.: conceptualization, methodology. D.G.: conceptualization, methodology, resources, funding acquisition, project administration, supervision, visualization, writing – original draft, writing – review and editing.

## Declaration of interests

D.G. is a co-founder and shareholder of primAAVera Therapeutics GmbH.

## Supplemental information

Document S1. Figures S1–S9

Table S1. Table containing the full dataset of periodicity assessment, related to Figures 2 and S2 Every row contains information on one replicate (roughly 1 × 10⁵ bp of sequence information) of sequences from an analyzed genus assessed for the periodicity of one specific dinucleotide.

Document S2. Article plus supplemental information
