# Supplementary material for: Distinct YY dinucleotide periodicity in adeno-associated virus DNA
Source: Mol Ther. 2026 Jul 8;34(9):5527–41. doi: 10.1016/j.ymthe.2026.07.003 (PMC13555545; doi:10.1016/j.ymthe.2026.07.003)
Supplement: Supplementary file 1 [file mmc1.pdf]

YMTHE, Volume *34*

Supplemental Information

Distinct YY dinucleotide periodicity

in adeno-associated virus DNA

Conradin Baumgartl, Jonas Becker, Lena Naber, Man Xu, Emma Gerstmann, Julie Garcia Gonzalez-Calero, Joyce Suppacher, Sebastian Heß, Felix Bubeck, and Dirk Grimm

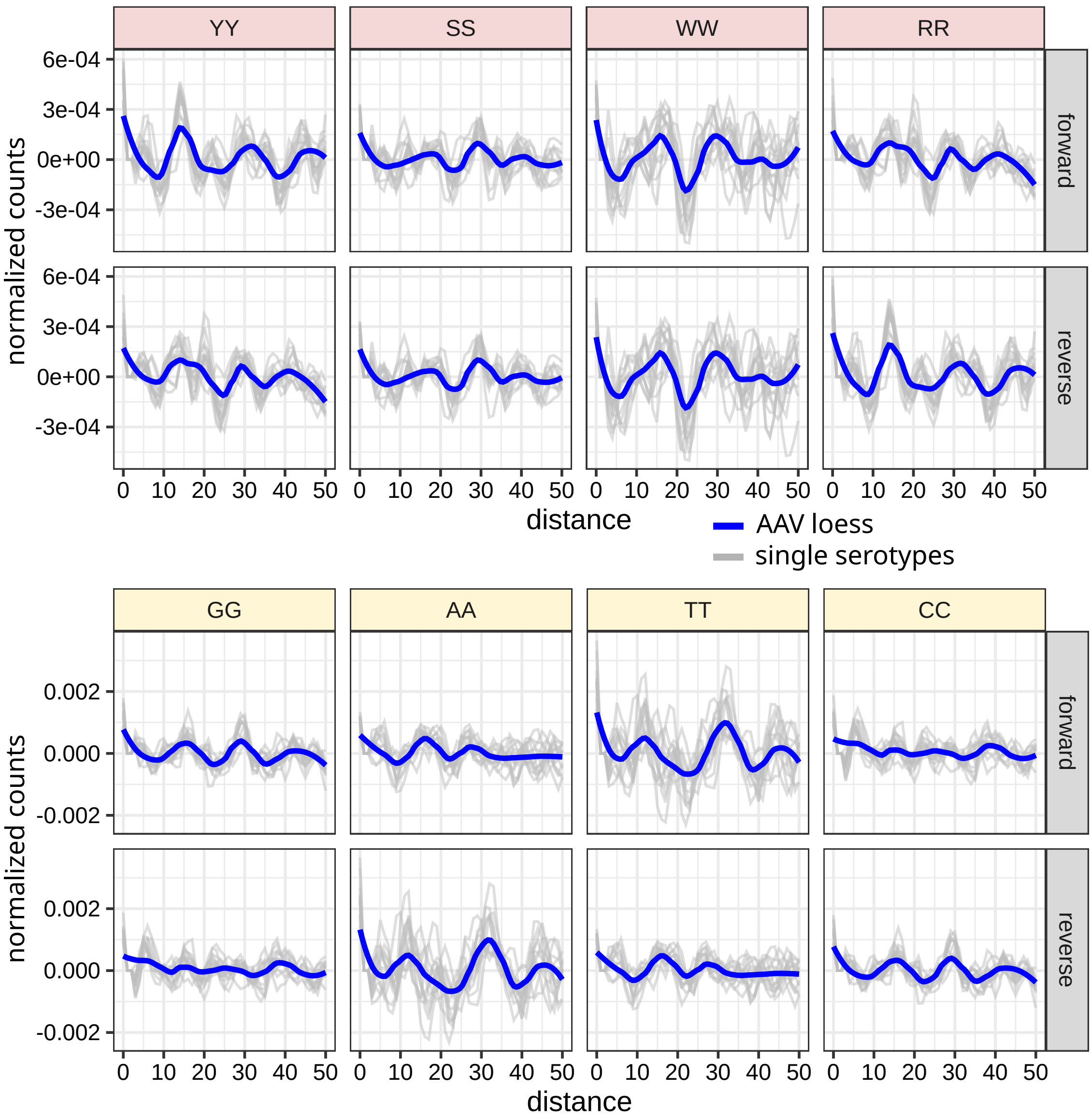

Figure S1. Normalized distance histogram of different dinucleotides and their combinations in the 13 primate AAV serotypes. Dinucleotide combinations and single dinucleotides are shown in the top and bottom panels, respectively.

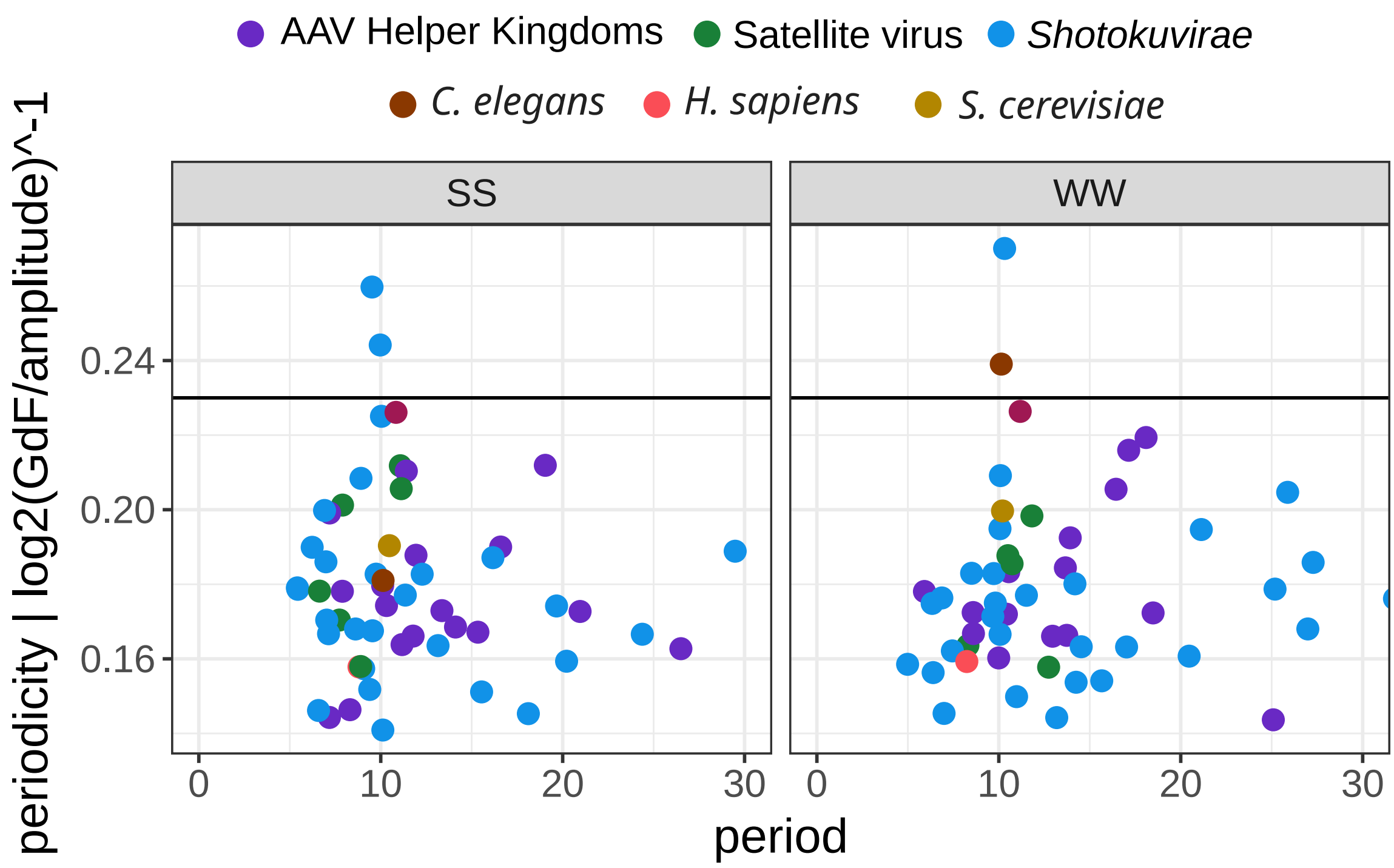

Figure S2. Periodicity of dinucleotides SS and WW of all analyzed sequence sets.

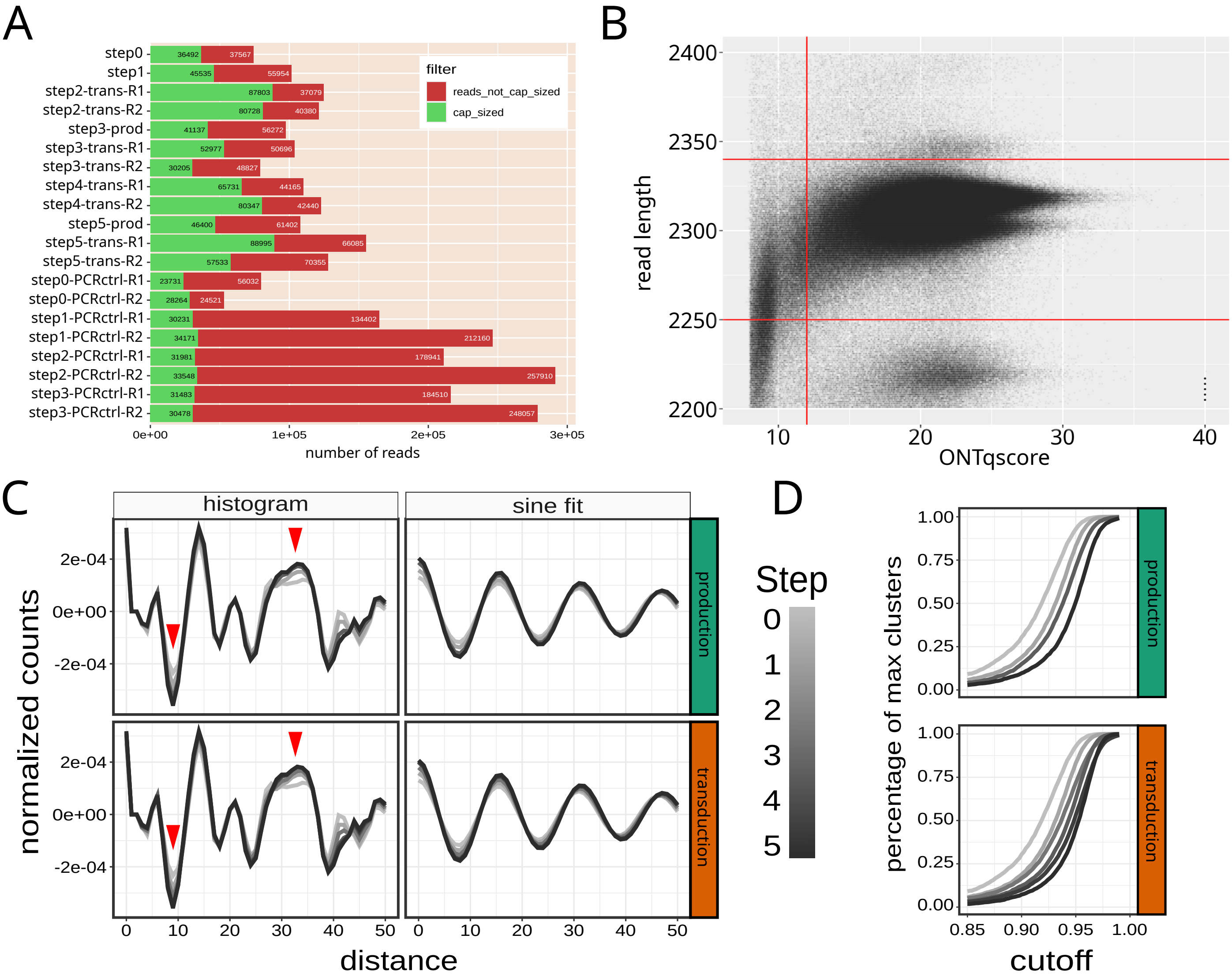

Figure S3. Quality control and detailed data on the selection of shuffled sequences. (**A**) Number of cap-sized reads retained after filtering. (**B**) Scatter plot depicting read length against quality score of an exemplary subset. The red lines indicate the quality of cutoff > 12 and the size restraint of 2250 bp < read_length < 2345 bp. (**C**) Normalized distance histograms and fitted sine curve of the production and transduction halves of the experiment. The red arrows indicate the deepening of the valley at ~9 bp and the increased definition of the peak at ~30 bp. (**D**) Cd-hit-est clustering while iterating over the clustering cutoff. The area under these curves was approximated for the estimation of diversity.

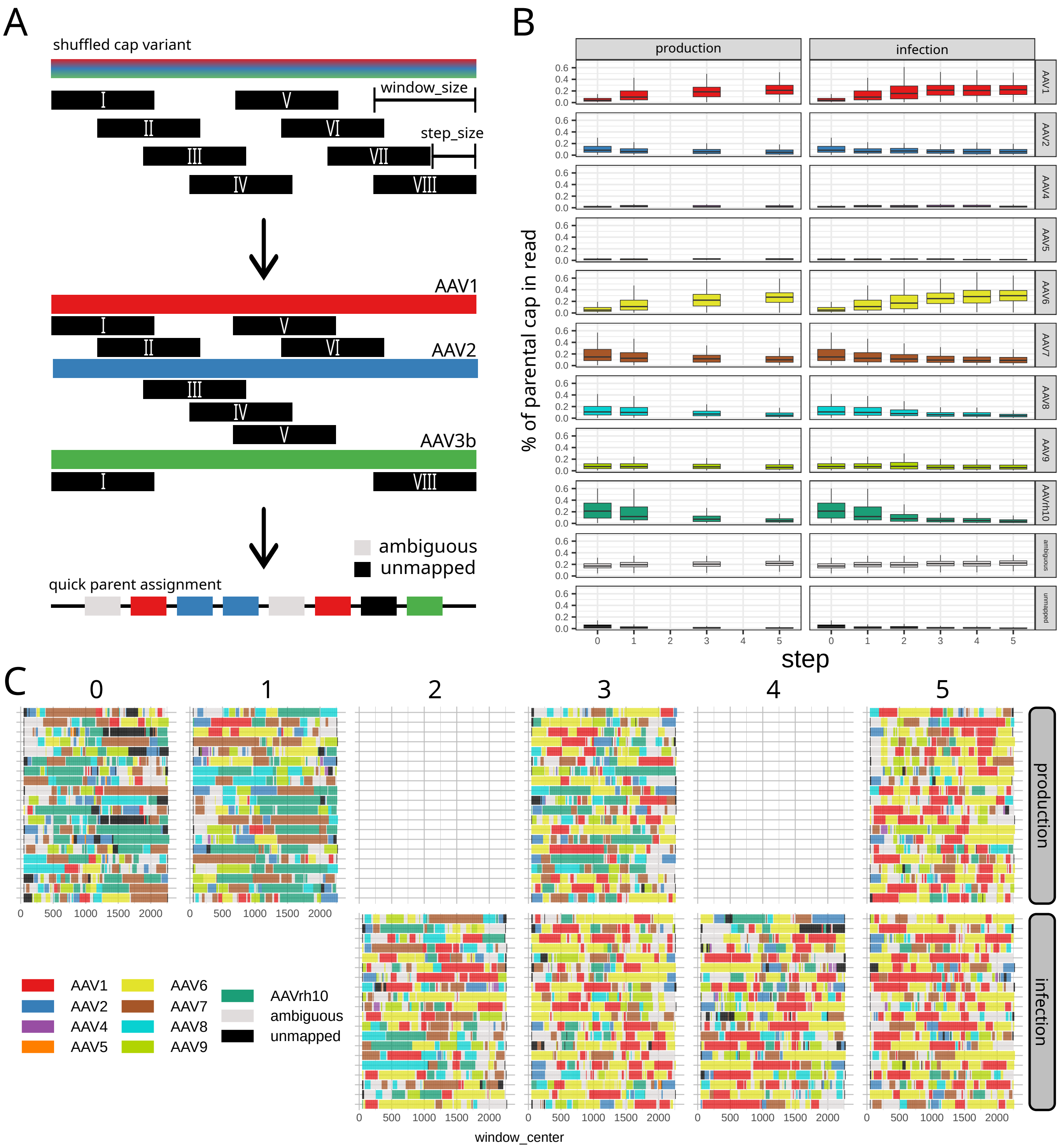

Figure S4. Quick Parent Assignment. (A) Schematic of how the parental assignment is conducted. A putative cap sequence is split using a sliding window approach and the resulting windows are individually aligned to a reference consisting of all parental sequences of interest. Window alignments are characterized as ambiguous if more than 1 alignment exists and the top hits do not differ more than 5 in Alignment Quality. (B) Percantages of each parental cap sequences in the obtained reads over the selection rounds in the production and transduction halves of the experiment. (C) Quick parental assignment of 20 random sequences from every experimental step.

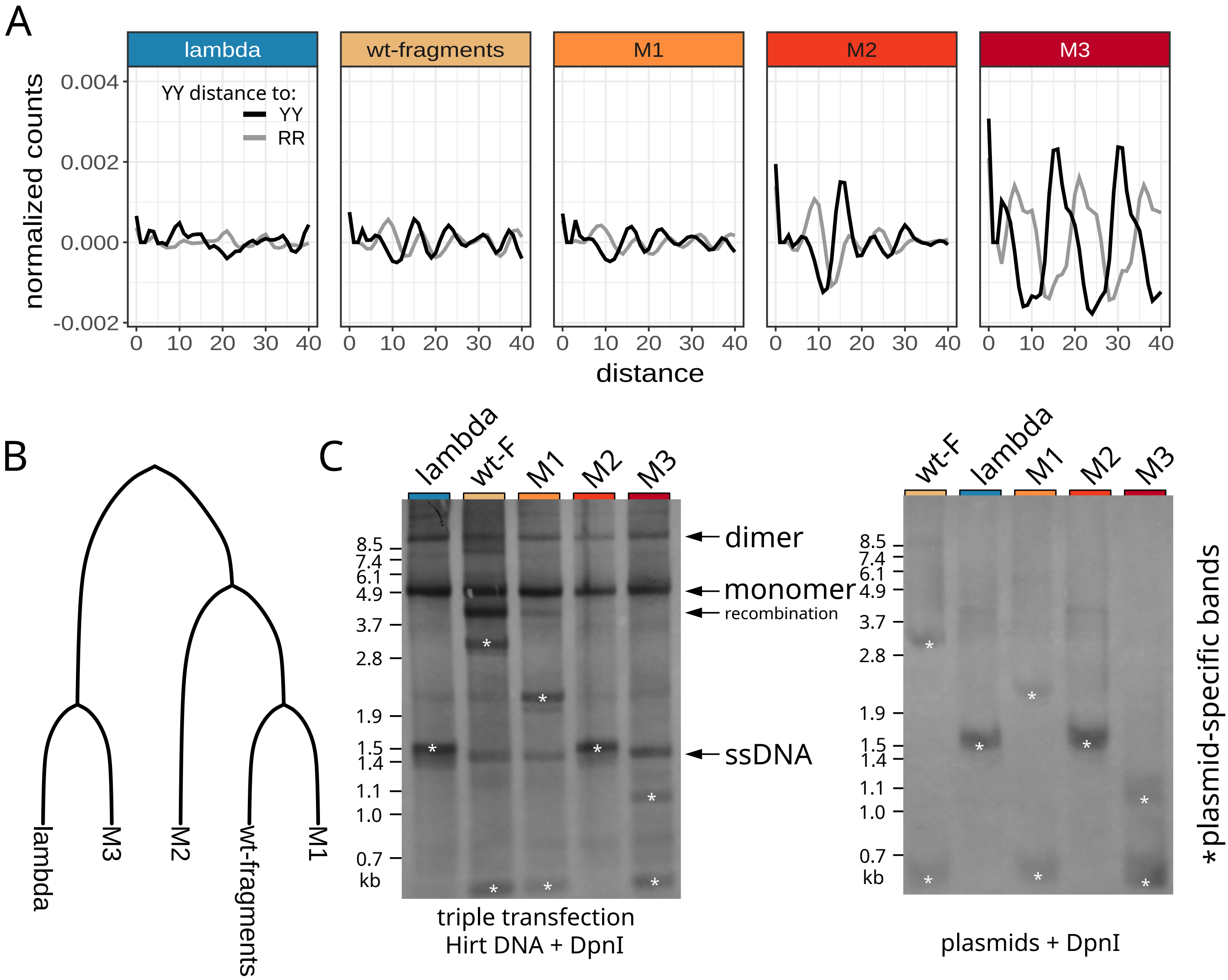

Figure S5. Supplementary information about the periodic stuffers. (A) Normalized distance histograms of the periodic stuffers, assessed using the same method as in Figure 1. (B) ClustalO clustering to show the similarity of the periodic stuffer sequences. (C) Southern blot of replication forms within Hirt DNA taken 3 days post-transfection in Hek293T cells. The left panel displays a Southern blot against a target within the promoter region of the transgene. The right panel shows the same Southern blot against plasmid negative controls. Plasmid-specific bands from the DpnI digestion are marked with asterisks.

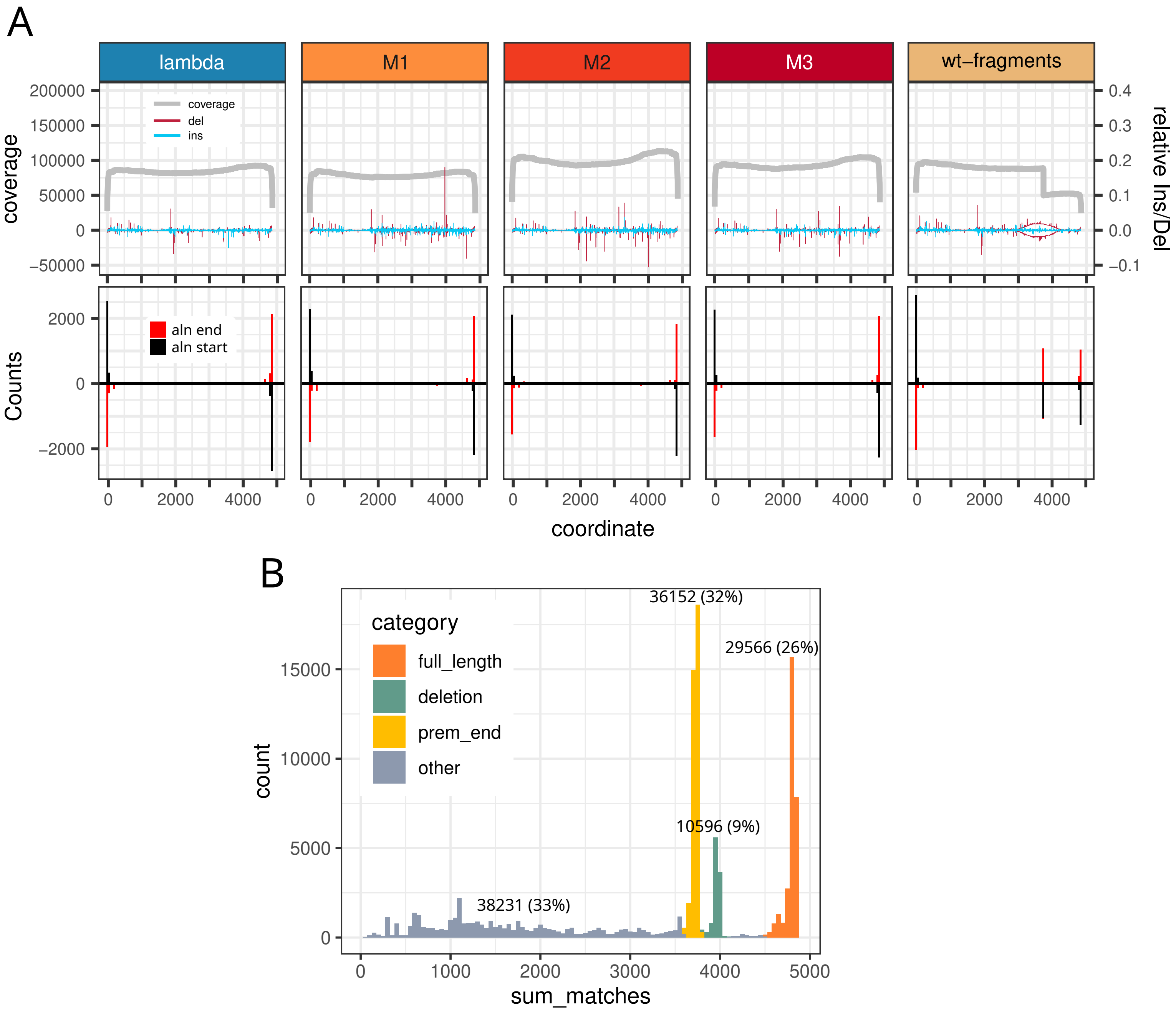

Figure S6. Genome truncation analysis from Nanopore sequencing of AAV vector productions. (A) Combined plot on alignment information. Top row shows total coverage and Insertion/Deletion rate across the transgene cassette reference. Insertion and Deletions are split by reference strand, with positive values indicating alignments to the forward strand and negative values alignments to the reverse strand. The bottom row is showing start and end positions of alignments, again split by forward and negative reference strand as before. (B) Histogram of the number of matches to the reference across all reads in the wt-fragments cassette vector (4.7 kb), with categories colored by full-length vectors, vectors with deletion, and vectors with a preliminary end of alignment.

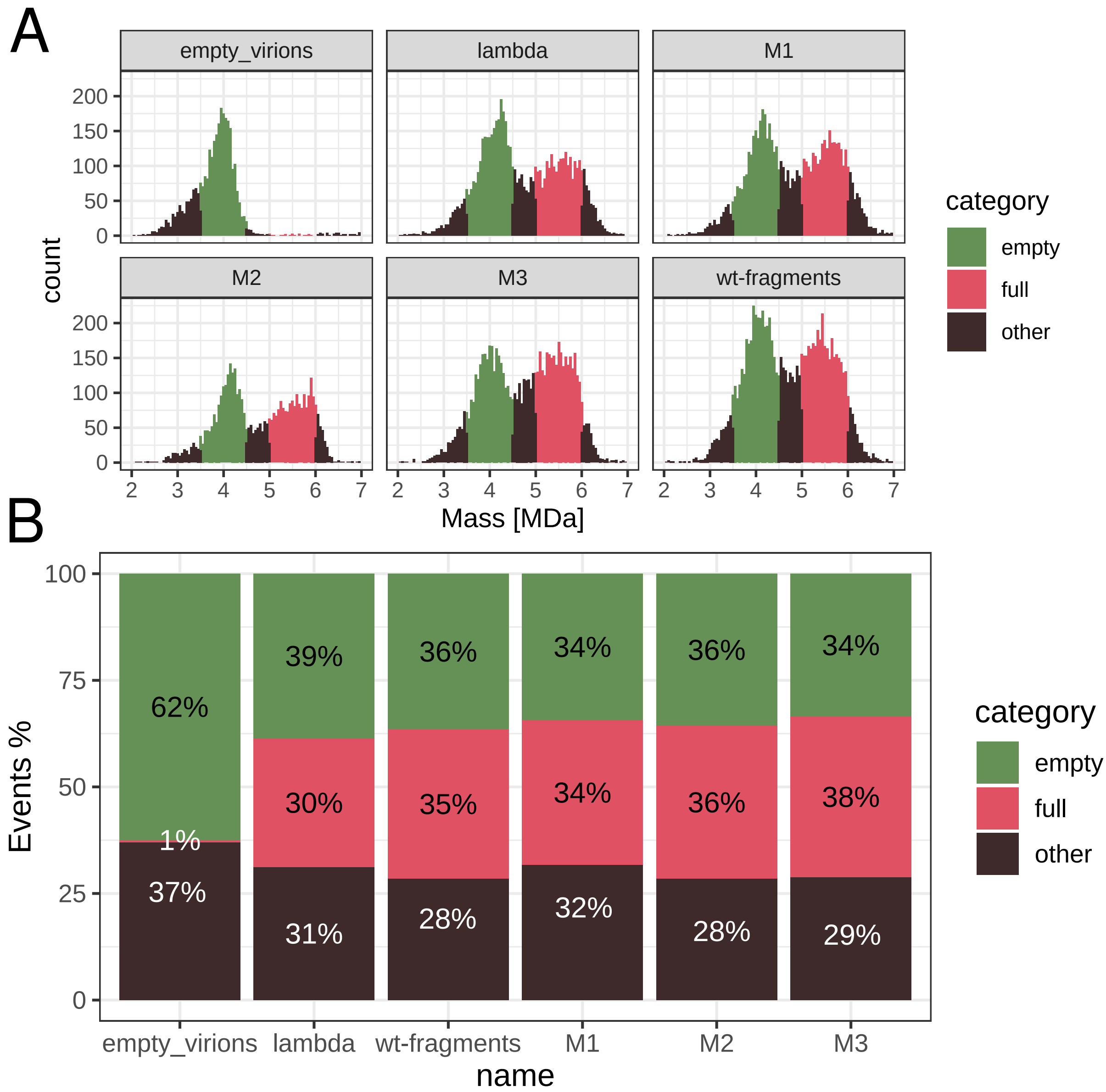

Figure S7. Full/empty ratio measured by mass photometry. (A) Histogram of masses of filtered events colored by their classification as empty of filled capsids. (B) Stacked barplot of percentages of the classes defined in A**.**

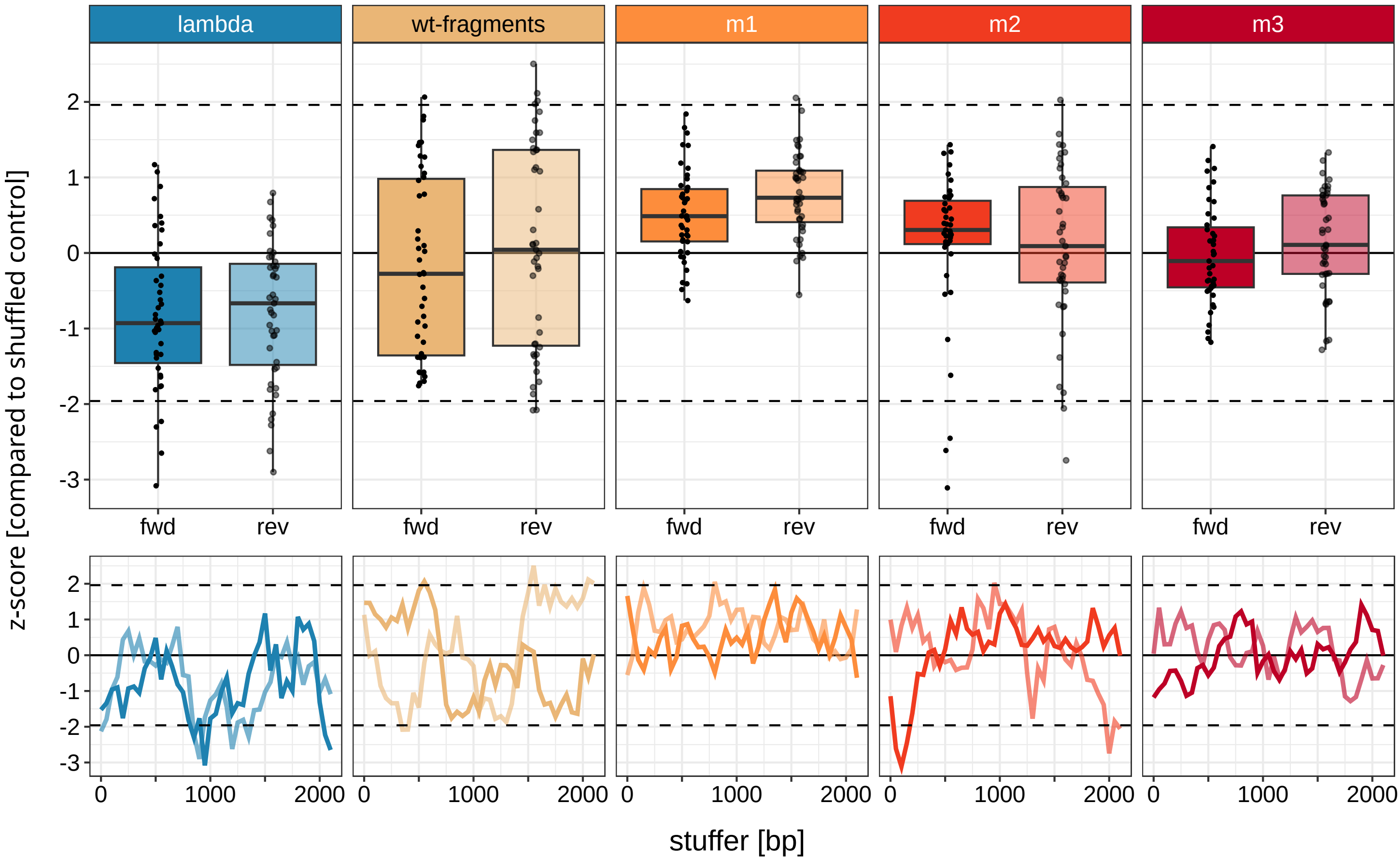

Figure S8. Ensemble free energy of the stuffer sequences measured in a sliding window approach normalized to a randomized control. The secondary structure formation of every window was interrogated with ViennaRNA for both forward and reverse orientations. The top row shows a boxplot of the z-score compared to the shuffled control across all windows. The bottom row shows the z-score across the length of the stuffer DNA. The z-score of 0 and the significance cutoff at +/- 1.96 are indicated.

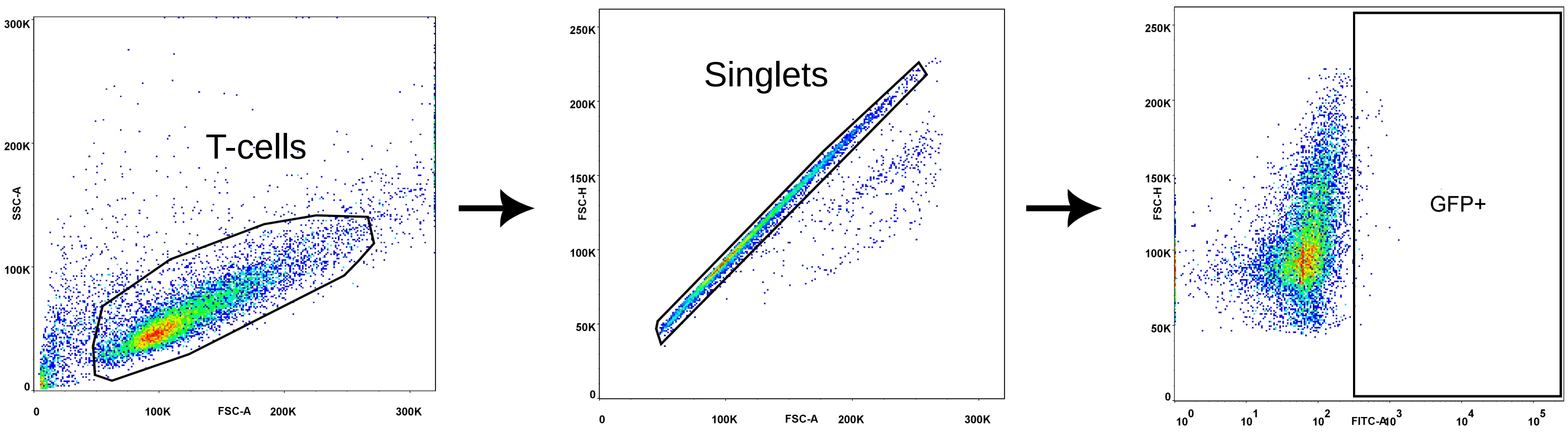

Figure S9. Gating strategy for GFP+ T-cell idenfitication. From left to right: total cell population was defined, singlets were gated, and GFP+ cells were identified in the FITC channel.

Table S1. Table containing the full dataset of periodicity assessment associated with Figures 2 and S2. Every row contains information on one replicate (roughly $1x10^5$ bp of sequence information) of sequences from an analyzed genus assessed for the periodicity of one specific dinucleotide.
